# Interplay between Thermal Denaturation and Dynamic Covalent Chemistry Generates High-Performance Recyclable Protein Plastics

**DOI:** 10.1021/acsmacrolett.6c00434

**Published:** 2026-10-01

**Authors:** Youwei Ma, Francesco Stellacci

**Affiliations:** † 27218 Institute of Materials, École Polytechnique Fédérale de Lausanne (EPFL), Lausanne 1015, Switzerland; ‡ Institute of Bioengineering, École Polytechnique Fédérale de Lausanne (EPFL), Lausanne 1015, Switzerland

## Abstract

Chemically recyclable and/or biobased polymers are promising alternatives to conventional petroleum-derived plastics, yet strategies that simultaneously improve their performance and recyclability remain scarce. Here, we report that the combined use of thermal denaturation and dynamic covalent chemistry can transform protein materials into strong, chemically recyclable bioplastics with enhanced hydrolytic stability. Cross-linking *a priori* brittle bovine serum albumin (**BSA**) with dynamic vinylogous urethane (**VU**) linkages produces robust plastics with tensile strengths of up to 35 MPa. Simultaneously, thermal denaturation at 80 °C restructures **BSA** from α-helices into more hydrophobic β-sheet-rich domains, reducing water absorption and thereby stabilizing the otherwise hydrolyzable **VU** cross-links. The resulting protein networks are chemically recyclable in acidic water, allowing the recovery of both **BSA** and cross-linkers. Overall, by exploiting the reciprocal interplay between dynamic covalent cross-linking and protein thermal denaturation, this work offers a fresh perspective to overcome the trade-off between polymer stability and recyclability.

## Graphical abstract

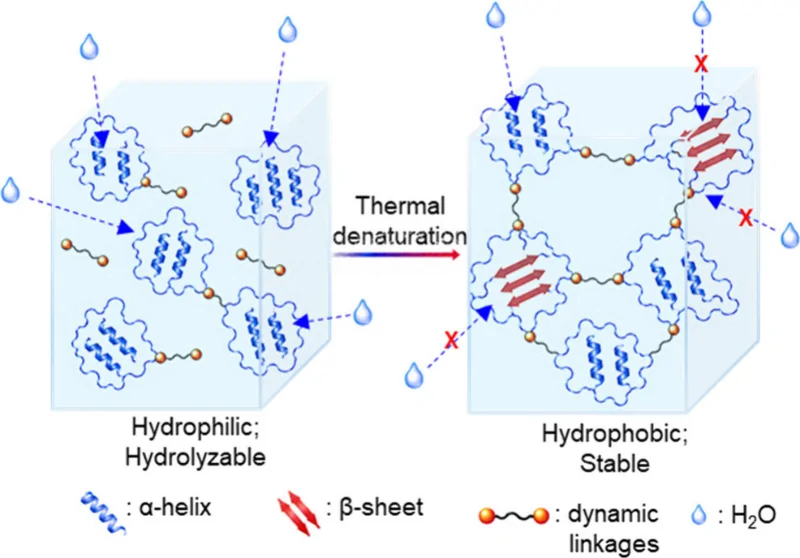

## Introduction

Developing sustainable plastics that combine high performance with circularity is essential for reducing reliance on fossil resources and catalyzing a circular material economy.^1,2^ Current strategies mainly rely on two approaches: the synthesis of chemically recyclable polymers through the incorporation of labile chemical motifs^3,4^ and/or the replacement of petroleum-derived feedstocks with a renewable biomass.^5–7^ The chemically recyclable polymers can be depolymerized into their constituent monomers/oligomers for reuse following their service life,^8^ while the biobased polymers benefit from renewable and biodegradable feedstocks.^9^

Despite their promise, both approaches face inherent limitations. For example, the labile chemical motifs, dynamic covalent bonds (**DCB**s) included, enable recycling of (postuse) thermosets, but their inherent susceptibility to degradation often leads to reduced stability, potentially compromising the polymer’s performance during regular use.^10,11^ Biomasses, such as cellulose, chitosan, lignin, and proteins, on the other hand, are abundant and renewable though, they are typically brittle because of their rigid molecular structures and strong intermolecular interactions.^5,7,12^ Common strategies to enhance their mechanical properties rely on the use of synthetic additives, coatings, cross-linkers or adhesives;^13–15^ while effective, they sometimes diminish the overall sustainability. More recently, cross-linking biomass with **DCB**s has emerged as a promising strategy, producing biobased dynamic covalent networks (**DCN**s) that exhibit enhanced service-life performance while remaining chemically recyclable or thermally reprocessable through dynamic bond dissociation or exchange.^12,16–18^ However, the resulting **DCN**s still inherit the intrinsic lability of **DCB**s.

Many efforts have been devoted to improving the stability of dynamic polymer networks, including the incorporation of mechanophores^19,20^ or supramolecular interactions such as hydrogen bonding,^21–23^ metal–ligand coordination,^24^ and hydrophobic interactions,^25^ or engineering phase-separation architecture.^26,27^ Although effective, these approaches generally increase molecular complexity and synthetic cost. Rather than introducing additional stabilizing motifs, we asked whether the inherent structural characteristics of the biomass itself could be exploited to address this issue. Proteins are particularly attractive in this regard because their hierarchical structures can undergo controlled conformational transitions.^28,29^ In their native state, proteins are rich in α-helices, whereas thermal denaturation disrupts the helical structure and forms more β-sheet-rich domains with increased hydrophobicity.^30^ We hypothesized that this increase in hydrophobicity could suppress water uptake and thereby improve the stability of hydrolyzable **DCN**s, whose **DCB**s—such as cyanurate,^31,32^ imine,^33–35^ tri/diketoenamines,^36–38^ vinylogous urethanes (**VU**s),^2,39–41^ and dioxaborolanes^42,43^ —are susceptible to depolymerization in aqueous media.

To test this concept, we developed thermally denatured protein networks by cross-linking bovine serum albumin (**BSA**) with poly­(ethylene glycol) bisacetoacetate (**aPEG**) at 80 °C, where thermal denaturation and dynamic cross-linking occur simultaneously (Scheme 1). The reaction between the acetoacetate groups of **aPEG** and the amino groups of **BSA** forms dynamic **VU** linkages, transforming intrinsically brittle protein films into strong bioplastics with tensile strengths of up to 35 MPa. Meanwhile, the protein thermal denaturation imparts good hydrolytic stability to the otherwise hydrolyzable **VU** networks (Scheme 1).

**Scheme 1:**
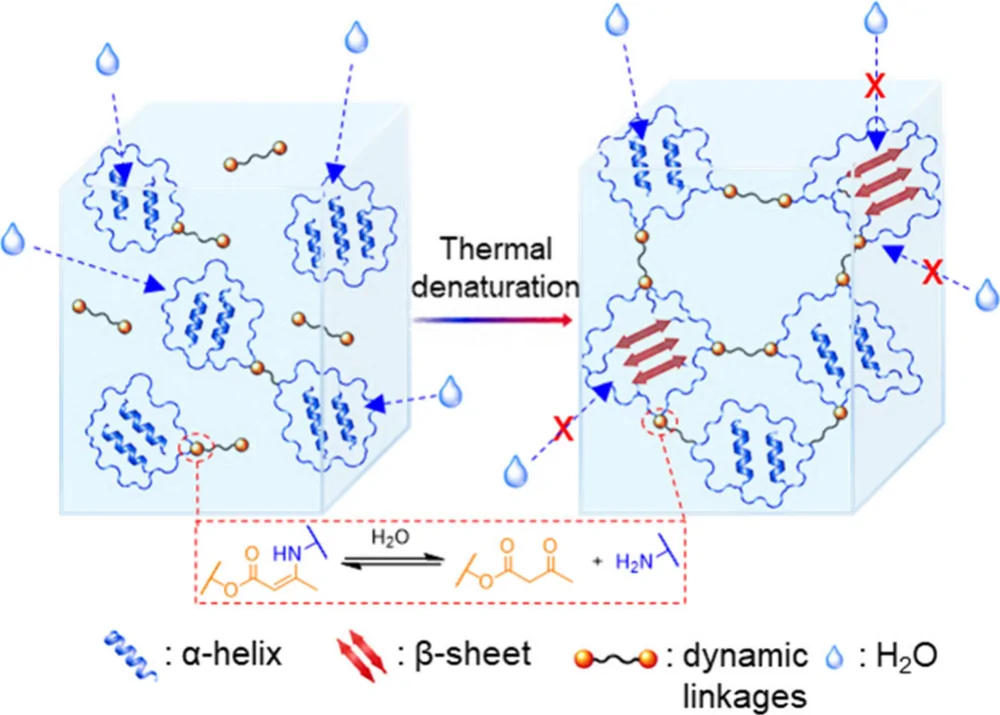
Illustration of the Thermal Denaturation of **VU**-Cross-Linked **BSA** Networks Leading to the Structural Transformation of the **BSA** Protein from Hydrophilic α-Helices to Hydrophobic β-Sheets, which Hinder the Hydrolysis of **VU** Linkages (Inside Dashed Square) Through Suppressing Water Uptake

**VU** bonds are generally formed through the condensation of acetoacetates and amines, accompanied by the generation of water as a byproduct.^41,44^ Condensation reactions are intrinsically reversible, thus according to the Le Chatelier’s Principle, the presence of water will bias the reaction backward, i.e., facilitating the hydrolysis.^45^ We first investigated the hydrolysis behavior of the **VU** motif by synthesizing two bis­(**VU**)-terminated compounds with opposite backbone polarity (Figure 1a); one is hydrophilic, comprising **PEG** as the core, and the other is derived from hydrophobic polypropylene glycol (**PPG**). Their preparation involved the initial acetoacetylation of **PEG** or **PPG** with *tert*-butyl acetoacetate (**tBA**), followed by the reaction of resulting **aPEG** or **aPPG** with *n*-butylamine (**Btl**) to produce **aPEG-Btl** or **aPPG-Btl**, respectively (Figures S1–S4 and S8–S11).

**Figure 1:**
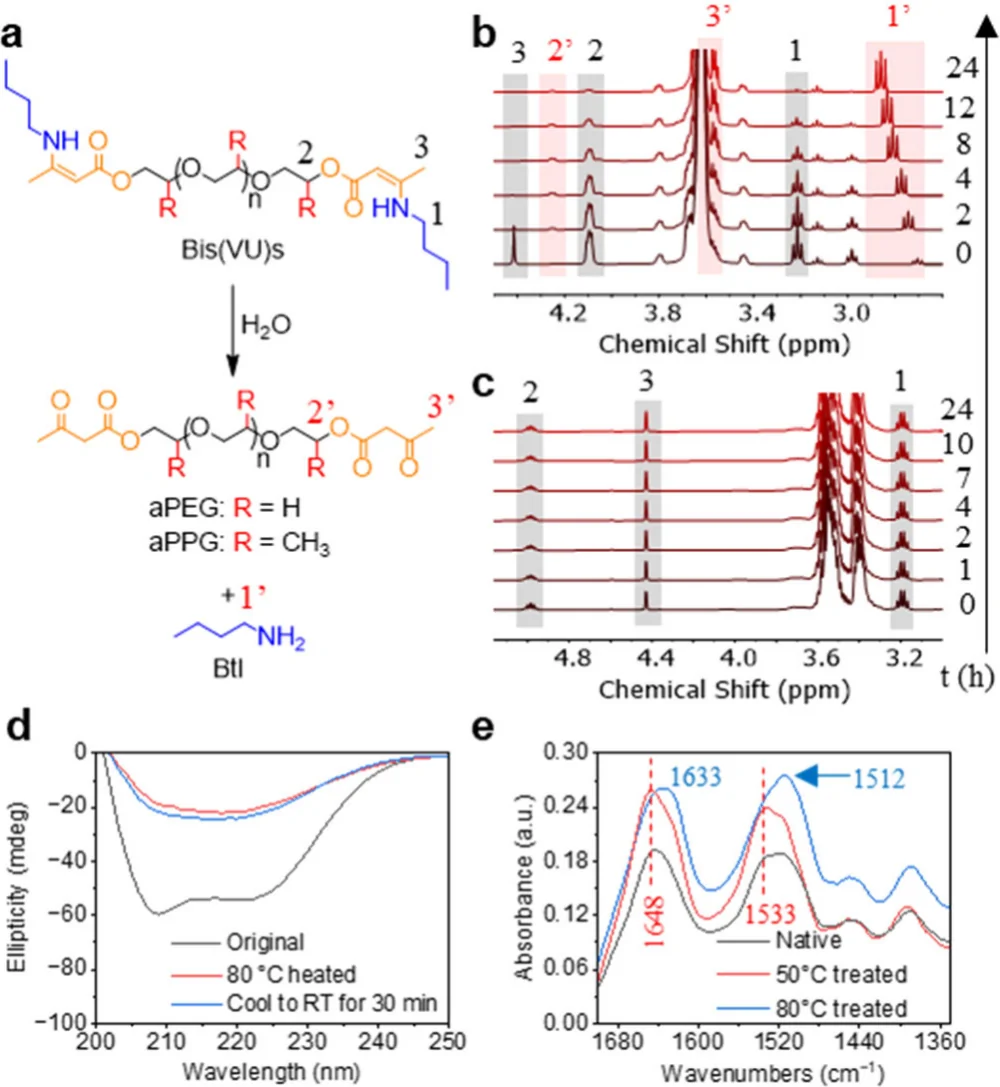
(a) Hydrolysis of bis­(**VU**)-terminated **PEG** or **PPG** (**aPEG**-/**aPPG-Btl**) into their respective **aPEG** or **aPPG** and **Btl**, with the assignment of the key protons shown in the ^1^H NMR spectra. Partial ^1^H NMR spectra recorded during the hydrolysis of (b) **aPEG-Btl** by D_2_O, or (c) **aPPG-Btl** in the presence of 1 M HCl–CDCl_3_ mixture solvent at 25 °C. (d) CD spectra of original **BSA** solution at a concentration of 10 μg mL^–1^, the solution after being heated at 80 °C for 2 h, and the heated solution cooled to room temperature for 30 min. (e) FTIR spectra of native **BSA** powder, solution-casted **BSA** films obtained at 50 and 80 °C.

In the case of **aPEG-Btl**, when mixing with excess D_2_O at 25 °C, ^1^H NMR analysis of the mixture shows that the signals assigned to **aPEG-Btl** weaken over time, alongside the appearance of the signals ascribed to **aPEG** and **Btl** (Figure 1b), confirming the hydrolysis of **aPEG-Btl** under such conditions. Integration of the ^1^H NMR signals reveals that the hydrolysis degree is 91% after 24 h at 25 °C, and increases substantially with increasing temperature (Figure S5). Reaching a similar conversion (i.e., ca. 90%) requires 6 h at 45 °C and only 1 h at 65 °C, which show complete hydrolysis after 9 and 3 h, respectively (Figure S5e). The hydrolysis reactions of **aPEG-Btl** (20 mg) proceeded in the presence of excess D_2_O (600 μL), thus falling under pseudo first-order condition. Plotting the consumption of **aPEG-Btl** as a function of time determined the kinetic rate constants (*k*), and subsequent Arrhenius model fitting of ln­(*k*) against 1/*T* afforded an activation energy (*E*_a_) of 60 kJ mol^–1^ for the reaction (Figure S6). Besides temperature, water content also influences the hydrolysis kinetics. Increasing the D_2_O volume from 400 to 800 μL increases the hydrolysis degree after 12 h from 74% to 81% (Figure S7). Together, these results demonstrate that hydrophilic **VU** compounds readily undergo hydrolysis in water, with the reaction kinetics governed by both temperature and water availability.

Unlike **aPEG-Btl**, **aPPG-Btl** is insoluble in water, and its hydrolysis was therefore investigated in a two-phase solution comprising CDCl_3_ and H_2_O or HCl (Figure S12b). **aPPG-Btl** dissolves in the CDCl_3_ phase, and while stirring, the aqueous phase can interact with the **VU** compound at the interface. ^1^H NMR analysis of the CDCl_3_ phase demonstrates negligible spectral changes over time, indicating that pure water is unable to hydrolyze the **VU** linkages (Figure 1c). In contrast, replacing the aqueous phase with 1 M HCl leads to gradual disappearance of the **VU** signals and regeneration of the starting materials, **aPPG** and **Btl** hydrochloride (Figure S12), confirming acid-catalyzed hydrolysis. The striking contrast between the hydrophilic and hydrophobic model compounds highlights the critical role of molecular polarity in regulating **VU** hydrolysis. Hydrophilic backbones facilitate water access to the dynamic linkages, leading to rapid hydrolysis, whereas hydrophobic environments effectively suppress water-induced bond cleavage while still allowing depolymerization under acidic conditions.

Next, protein thermal denaturation was explored using **BSA** as a model given its abundance and low cost. Circular dichroism (CD) spectroscopy of native **BSA** displays two characteristic negative bands at 208 and 222 nm (Figure 1d), attributed to its rich α-helix structures.^46^ Upon thermal treatment at 80 °C for 2 h, the two bands merge into a broad minimum centered at 216 nm ascribed to β-sheet-rich conformations, which remains essentially unchanged after cooling to room temperature (Figure 1d). These phenomena indicate that **BSA** undergoes denaturation at 80 °C, affording more β-sheets at the loss of some α-helices in an irreversible manner. Previous studies demonstrate that the irreversible change is attributed to the synergistic efforts of intermolecular disulfide formation, hydrophobic interactions and intermolecular β-sheet association.^47,48^ The formation of more β-sheets induced by thermal treatment increases the average hydrodynamic diameter (*D*) of **BSA** in water for 2.4 folds from 10.4 to 25.3 nm, as determined by Dynamic Light Scattering (DLS) (Figure S13). The results coincide with Fourier Transform Infrared (FTIR) analysis (Figure 1e); With increasing treatment temperature, the characteristic C=O stretching and N–H bending bands of α-helices at 1648 and 1533 cm^–1^ gradually decrease, accompanied by the emergence of new bands at 1633 and 1512 cm^–1^ corresponding to aggregated β-sheet structures (Figure 1e).^49^ These observations collectively confirm the progressive thermal denaturation of **BSA** and the formation of β-sheet-rich domains. The thermal denaturation at 80 °C is significant, and the treated **BSA** solution underwent gelation and was phase-separated from the aqueous medium (Figure S14a, right), suggesting increased hydrophobicity. By comparison, the sample treated at 50 °C remained as a homogeneous solution (Figure S14a, left). Consistent with this observation, the film prepared at 80 °C exhibits a higher water contact angle (85°) than that (48°) obtained at 50 °C (Figures S14b,c), and also absorbs less moisture in a humid environment (75%RH) compared to the nonheated **BSA** (Figure S14d). Clearly, thermal denaturation disrupts the native α-helical structure of **BSA**, and exposes hydrophobic residues, giving rise to β-sheet-rich aggregated structures with enhanced hydrophobicity that suppresses water ingress.

Having investigated the nature of **VU** bonds and **BSA** proteins, we set out to prepare protein networks (**BSA-xaPEG**) through cross-linking **BSA** with **VU** linkages using **aPEG** as the cross-linker (Figure 2a). Parameter **x** represents the weight ratio of **aPEG** to **BSA** as 10, 20, and 30 in percentage. After the cross-linking at 80 °C, the disappearance of the peaks of the acetoacetate groups at 1745 and 1716 cm^–1^ and the appearance of two new peaks at 1645 and 1605 cm^–1^ in the FTIR spectrum of **BSA-20aPEG** supports the consumption of acetoacetates to form **VU** linkages through the reaction with the amino functionality of **BSA** (Figure S15). Swelling experiments show that the cross-linked networks maintain their structural integrity in H_2_O and they exhibit decreased swelling ratios from 610% to 455% upon increasing **x** from 10 to 30 (Figure S16).

**Figure 2:**
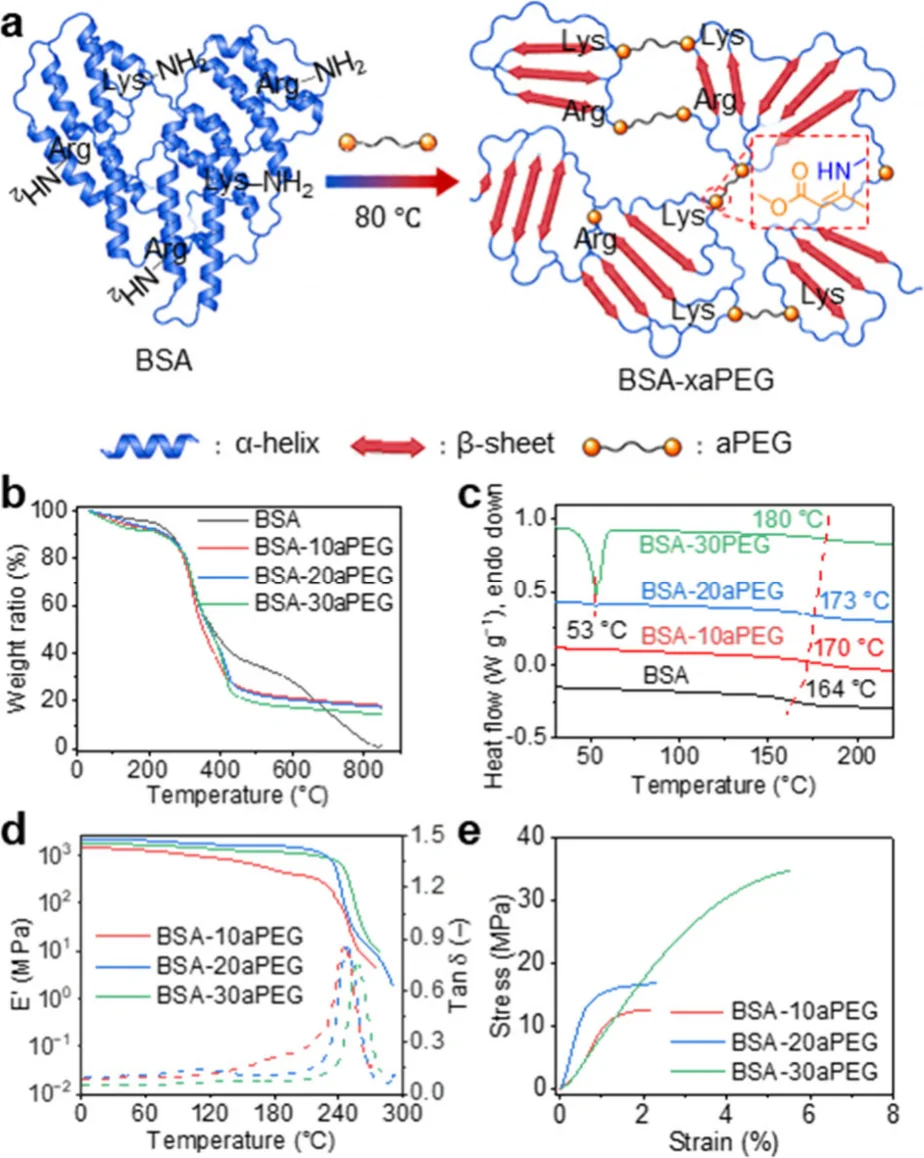
(a) Illustration of the preparation of **BSA-xaPEG** through cross-linking **BSA** with **aPEG** at 80 °C, with the possible cross-linking sites on the residues of arginine (Arg) and lysine (Lys). (b) Thermogravimetric curves, (c) DSC traces, (d) DMA traces (*E*′ in solid lines and tan δ in dashed lines), and (e) stress–strain curves of **BSA** and **BSA-xaPEG** films. **BSA** film is brittle and nonmeasurable in DMA and tensile testing analyses.

**BSA-xaPEG** films display good thermal stability and start to decompose at a high temperature of above 250 °C thanks to the strong intra- and intermolecular interactions of **BSA** proteins (Figure 2b). Moreover, the incorporation of cross-link architecture increases the char ratio from 3% of **BSA** to ca. 20% of **BSA-xaPEG**, measured at 800 °C (Figure 2b). The thermal properties of the films were further explored by Differential Scanning Calorimetry (DSC). It shows that **BSA** has a glass transition temperature (*T*_g_) of 164 °C. The cross-linking with **aPEG** leads to increased *T*_g_ first to 170 °C of **BSA-10aPEG**, then to 173 °C of **BSA-20aPEG** and 180 °C of **BSA-30aPEG** (Figure 2c), due to the restricted chain mobility.^50^ In addition, **BSA-20aPEG** and **BSA-30aPEG** exhibit an additional endothermic peak at 53 °C, which is attributed to the melting of crystalline **PEG** segments in the films (Figure 2c).

The (thermo)­mechanical properties of the **BSA-xaPEG** films were subsequently studied by Dynamic Mechanical Analysis (DMA) and tensile testing. The pure **BSA** films, regardless of undergoing thermal denaturation at 80 °C or not, were too brittle to permit reliable mechanical characterization. DMA traces of all three protein films show a sharp drop in storage modulus (*E*’), accompanied by a peak in tan δ plots at the temperature of 242–255 °C (Figure 2d), indicative of their *T*_g_. The *T*_g_ values increases with **aPEG** content, mirroring the trend found in the DSC analysis (Figure 2c). Even though a small peak at ca. 100 °C was noted in tan δ plots of both **BSA-20aPEG** and **BSA-30aPEG** films, associated with the melting temperature of their **PEG** domain, this thermal transition does not lead to significant decline in *E*’ (Figure 2d). This indicates the tiny impact of the crystalline zone on the thermomechanical properties of the films. The increase of **aPEG** content, marking the increased cross-link density, increases both stress and strain at break of **BSA-xaPEG** samples (Figure 2e). Particularly, when the **aPEG** content increases from 20 wt % to 30 wt %, obvious increases from 17 MPa and 2% to 35 MPa and 6% in stress and strain at break, respectively, are noted (Figure 2e). This pronounced improvement might arise from enhanced network connectivity, along with more efficient stress transfer brought by more flexible **PEG** bridges.^41^ This is rather rewarding given that it is not possible to determine the (thermo)­mechanical properties of pure **BSA** film with the same measurement efforts. Neither are previous studies, to our knowledge, that have reported the tensile behavior of pure **BSA** films due to their high brittleness, and most of them used **BSA** as (hydro)­gels or reinforcing fillers through blending with other polymers or plasticizers (e.g., water and polyol).^51–56^ As a consequence, the plasticized proteins usually show low tensile strength of less than 10 MPa depending on the type and the content of plasticizers,^57–59^ whereas blending proteins with mechanically robust polymers or reinforcing phases can increase their strength, in some cases to several tens of megapascals.^60^ The above results show that the incorporation of cross-linking structure is effective in improving the performance of **BSA** proteins.

The impact of water on the mechanical properties of **BSA-xaPEG** films was also explored by immersion of **BSA-20aPEG** in water. Upon immersion in water, the film rapidly absorbs water and softens within minutes, accompanied by a sharp transition from strong and stiff to soft and stretchable (Figure S17). After only 5 min in water, the stress at break decreases from 17 to 1.4 MPa, while the strain at break increases from 2% to 69% (Figure S17). Such rapid water-responsive softening may enable applications in actuators^61^ or facial masks where low modulus and the biobased nature of the material are desirable.^62^

Last, we studied the influence of thermal denaturation on the recyclability of the protein films. We selected **BSA-20aPEG** as the model film, and to do comparison, a control sample was prepared in the same composition but at a lower temperature of 50 °C. Such a temperature is sufficient to promote **VU** formation between **BSA** and **aPEG** while not to induce extensive denaturation (Figure S14).^2^ FTIR analysis demonstrates that the control sample shows relatively weaker β-sheet-associated peaks at 1633 and 1512 cm^–1^ compared to the **BSA-20aPEG** film, obtained at 80 °C (Figure S18a). The 80 °C-treatment also imparts higher hydrophobicity, and affords the film with a water contact angle of 93° in comparison to only 63° for the control sample (Figures S18b,c). The less-denatured control film is susceptible to hydrolysis and can dissolve in deionized water (Figure S19a), indicating the poor hydrolytic stability. Leveraging the hydrolysis-induced decross-linking, the constituent materials including **BSA** and **aPEG** were recovered in high quality, as confirmed by FTIR and ^1^H NMR measurements (Figures S19b,c).

By contrast, **BSA-20aPEG** remains integral in pure water solution (Figure S16a) and suggests good water resistance. This primarily results from the increased hydrophobicity induced by thermal denaturation (Figure S18). To enable the recycling of the hydrophobic **VU** polymer networks, according to the knowledge that we gained in the above small-molecule model study (Figure S12), acidic solution was adopted (Figure 3a). Following decross-linking in acid and a workup to separate the depolymerized mixture, **BSA** was first recovered in 89% yield, with its structure and thermal decomposition behavior compared with the denatured **BSA** treated at 80 °C (BSA-80); no significant difference is noted in both the FTIR spectra and TGA curves (Figures 3b, S20). However, when we further analyzed these **BSA** films by simultaneous wide- and small-angle X-ray scattering (SAXS/WAXS) experiments, the broad scattering maxima of BSA-80 at q* = 0.66 and 0.2 A^–1^, associated with α-helix-related packing and interprotein correlations, respectively, shift to 0.6 and 0.11 A^–1^ after the acid-assisted recycling (Figure S21).^63,64^ This is probably due to conformational and intermolecular expansion upon protonation in the recycling.^65^ Neutralization of the recovered **BSA** with 0.1 M NaHCO_3_ aqueous solution restores both scattering maxima to positions close to those of BSA-80 (Figure S21).^66^ The above findings indicate that **BSA** component could be recovered in high quality.

**Figure 3:**
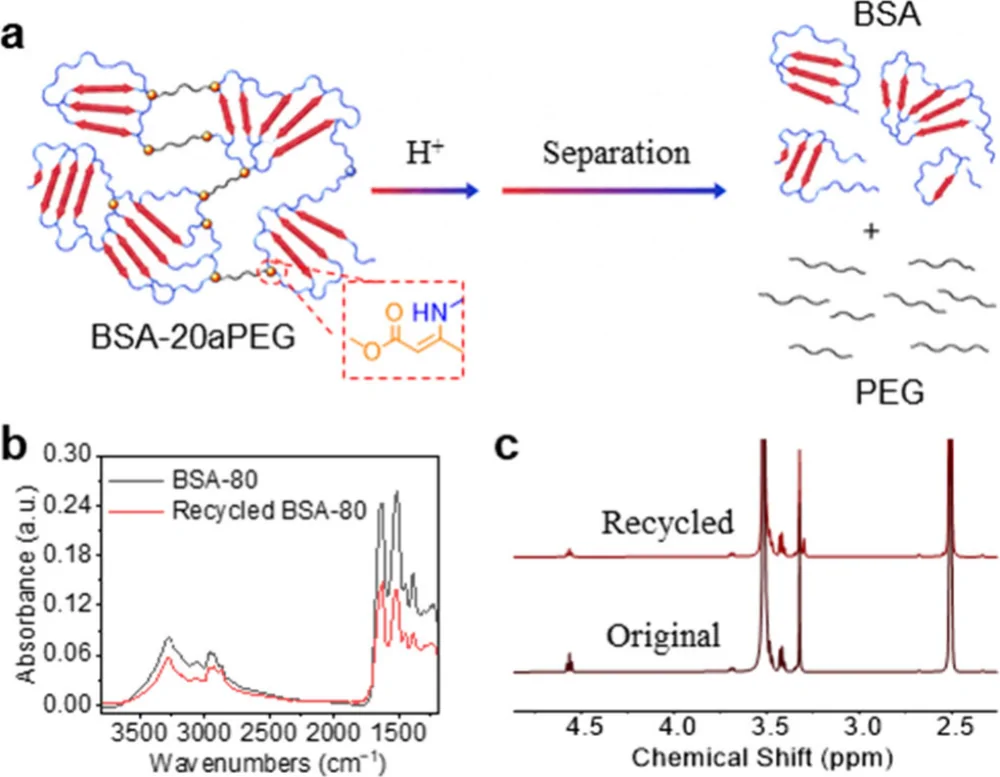
(a) Illustration of the acid-catalyzed hydrolysis of **BSA-20aPEG**, followed by chemical separation to recover **BSA** and **PEG**. Comparisons of (b) FTIR spectra between 80 °C-treated and the recycled **BSA**, and (c) ^1^H NMR spectra of original and recycled **PEG**.

To our surprise, analysis of the other component reveals a complete loss of the acetoacetate groups, affording **PEG** in 83% yield instead of the original **aPEG** (Figure 3c). Subsequent investigation of this phenomenon using NMR spectroscopy indicates that the terminal acetoacetate groups possibly undergo decarboxylation under acidic conditions (Figure S22), which resonates with our previous finding.^67^

## Conclusion

In summary, we have found a reciprocal interplay between hydrolyzable dynamic covalent bonds (**DCB**s) and protein thermal denaturation. Dynamic covalent cross-linking transforms intrinsically brittle protein materials into robust bioplastics with improved mechanical performance, while thermal denaturation of the protein matrix simultaneously stabilizes the otherwise hydrolyzable **DCB**s by suppressing water ingress through the formation of hydrophobic β-sheet-rich domains. This synergistic relationship enables the resulting protein networks to combine high performance, enhanced hydrolytic stability, and chemical recyclability. More broadly, our strategy exploits the intrinsic structural evolution of biomass, rather than introducing additional chemical alterations, to regulate the behavior of dynamic polymer networks. We anticipate that this concept can be readily extended to other hydrolyzable dynamic covalent chemistries and biomass feedstocks, providing a general design principle for the development of high-performance yet recyclable bioplastics.

## Supplementary Material



## Notes

Additional experimental details, materials, and methods, including photographs of experimental setup (PDF)
