# Supplementary material for: Interplay between Thermal Denaturation and Dynamic Covalent Chemistry Generates High-Performance Recyclable Protein Plastics
Source: ACS Macro Lett. 2026 Oct 1;15(10):1558–64. doi: 10.1021/acsmacrolett.6c00434 (PMC13689914; doi:10.1021/acsmacrolett.6c00434)
Supplement: Supplementary file 1 [file mz6c00434_si_001.pdf]

Support Information

for

# The Interplay between Thermal Denaturation and Dynamic Covalent Chemistry Generates High-Performance Recyclable Protein Plastics


Youwei Ma,[1*] Francesco Stellacci[1,2*]

1. Institute of Materials, École Polytechnique Fédérale de Lausanne (EPFL) Lausanne 1015, Switzerland

2. Institute of Bioengineering, École Polytechnique Fédérale de Lausanne (EPFL) Lausanne 1015, Switzerland

*Correspondence: Y. Ma, youwei.ma@epfl.ch; F. Stellacci, francesco.stellacci@epfl.ch

# Contents

Materials and Instrumentation .......... 2

Materials .......... 2

Instrumentation .......... 2

Synthesis, Characterization and Recycling .......... 3

Synthesis and hydrolysis of bis(vinylogous urethane)-terminated polyethylene glycol (PEG).......... 3

Synthesis and hydrolysis of bis(vinylogous urethane)-terminated polypropylene glycol (PPG) ..... 11

Synthesis and recycling of VU-cross-linked bovine serum albumin (BSA-aPEG) .......... 16

## Materials and Instrumentation

### Materials

Poly(ethylene glycol) (**PEG**, $M_n$ = 2000 g mol$^{-1}$), polypropylene glycol (**PPG**, $M_n$ = 2000 g mol$^{-1}$), bovine serum albumin (**BSA**, 98%), *tert*-butyl acetoacetate (98%, **tBA**), *n*-butylamine (99.5%, **Btl**), chloroform-*d* (99.8%, $CDCl_3$), DMSO-$d_6$ (99.9%), deuterium oxide (99.9%, $D_2O$), tetrahydrofuran (99.9%, THF), chloroform (99.8%), and ethanol (98%) were purchased from Sigma–Aldrich. All chemicals were used as received.

### Instrumentation

**Nuclear Magnetic Resonance (NMR) spectroscopy.** NMR spectroscopy was carried out at 297.2 K on a Bruker Avance DPX 400 spectrometer at frequencies of 400.19 MHz for $^1$H nuclei and 100.63 MHz for $^{13}$C nuclei. Spectra were calibrated to the residual solvent peak of $CDCl_3$ (7.26 ppm $^1$H NMR; 77.16 ppm $^{13}$C NMR), DMSO-$d_6$ (2.50 ppm $^1$H NMR; 39.52 ppm $^{13}$C NMR), and $D_2O$ (4.79 ppm $^1$H NMR). Data were evaluated with the MestReNova software suite (v 12.0) and all chemical shifts (δ) are reported in parts per million (ppm) relative to tetramethylsilane with coupling constant (J) in Hz (multiplicity: s = singlet, d = doublet, t = triplet, q = quartet, m = multiplet, dd = double doublet).

**Fourier Transform Infrared (FTIR) Spectroscopy**. FTIR was carried out on a Bruker Tensor 27 spectrometer. Samples were analyzed in attenuated total reflectance (ATR) mode using a diamond crystal.

**Dynamic Light Scattering (DLS) measurement**s were performed in stable dilute protein solution with a concentration of 2 mg mL$^{-1}$ at room temperature (ca. 25 °C). The scattering angle is 173°, and each sample was measured for three times, in which the sample was scanned for 13 times during every measurement.

**Thermogravimetric Analyses (TGA)** were performed with a Mettler–Toledo TGA/DSC 1 Stare System. The temperature ranged from 25 °C to 850 °C with a heating rate of 10 °C min$^{-1}$. Tests were carried out under nitrogen with a flow rate of 40 mL min$^{-1}$. TGA results were analyzed using the STARe Evaluation software.

**Differential Scanning Calorimetry (DSC) measurements** were performed under a nitrogen atmosphere using a Mettler–Toledo STAR system operating at a heating/cooling rate of 10 °C min$^{-1}$ in the temperature range of 25 to 225 °C using a sample mass of ca. 5 mg. The melting temperature, $T_m$, is reported based on the minimum of the major endothermic melting peak.

**Dynamic Mechanical Analyses (DMA)** were performed on the TA Instrument Model Q800 in tension mode. The temperature ranged from 0 to 280 °C with a heating rate of 5 °C min$^{-1}$, a frequency of 1 Hz, and an amplitude of 0.05% strain.

**Tensile testing.** If not indicated, stress–strain measurements were carried out at 20 mm $min^{-1}$ with a Zwick/Roell Z010 tensile tester equipped with a 50 N load cell. Uniaxial tensile tests were carried out with dog-bone-shaped samples with dimensions of 40 × 5 × 0.2 mm (length × width × thickness) that were cut from the solution-casted films according to ASTM D1708. The testing procedure followed the standard method described in ASTM D1708.

**Solubility experiment.** The weighed samples ($M_{initial}$) were immersed in deionized water and shaken in a shaking bed for 24 h. The water was changed every 8 h. The solvent was carefully removed with a syringe, and the surface was wiped with a tissue after swelling, after which the samples were weighed ($M_{swelling}$). The swelling ratio was defined as $M_{swelling}/M_{initial}$.

**Circular Dichroism (CD) Analysis.** The CD spectra were recorded on a Jasco J-1500 CD Spectrometer from 200 to 250 nm, using a data pitch of 0.2 nm, a bandwidth of 1 nm, and a scan speed of 20 nm $min^{-1}$. The protein samples were prepared to a concentration of 10 µg $mL^{-1}$ and measured in a 0.1 cm pathlength Hellma quartz cuvette. The sample solution was initially measured at 25 °C, and subsequently heated at 80 °C for 2 h for the second measurement, followed by cooling to 25 °C again for 30 min before the third measurement.

**Water contact angle measurements** were performed on a contact angle analyzer (Krüss Easy Drop, Germany) with an injection volume of 2 μL. The image was taken upon contact with a water droplet for 10 s.

**Small-angle X-ray scattering (SAXS) and wide-angle X-ray scattering (WAXS) analyses** were conducted on Xenocs Xeuss 3.0 equipped with a Cu microsource (wavelength Cu-Kα 1.5406 Å) and a Dectris Eiger 1M detector (Xenocs, France). The sample-to-detector distance was 100 mm. The measurements were performed under vacuum at room temperature. The exposure time of the samples to the X-ray source was set as 5 min. The 1D *I(q)* spectra of the film were background subtracted from a spectrum *I(q)* of the empty holder. No normalization to the film thickness was made.

## Synthesis, Characterization and Recycling

### Synthesis and hydrolysis of bis(vinylogous urethane)-terminated polyethylene glycol (PEG)

**Synthesis of PEG bisacetoacetate (aPEG)**. As shown in Figure S1, commercial **PEG** (20 g, 10 mmol) with an $M_n$ of 2000 g $mol^{-1}$ was weighed and added into a 100 mL single-necked flask, together with a stirring bar. Then, *tert*-butyl acetoacetate (**tBA**, 10 equiv.) was added in one portion with a pipette. The flask was equipped with a condenser and a Dean–Stark apparatus to remove the *tert*-butanol generated during the reaction. The resulting mixture was heated at 155 °C for 3 h. The unreacted **tBA** was removed by vacuum distillation at 100 °C for 12 h to afford **aPEG** (21.1 g; yield 96%).

$^{1}H$ NMR (400 MHz, DMSO-$d_6$) δ 4.22 – 4.14 (m, 2H), 3.64 – 3.58 (m, 4H), 3.51 (s, 87H), 2.18 (s, 3H).

**Synthesis of bis(vinylogous urethane)-terminated PEG**. As shown in Figure S1, in a 25 mL two-necked flask equipped with a reflux condenser and a magnetic stir bar, butylamine (**Btl**; 0.73 g, 10 mmol) was combined with **aPEG** (2.2 g, 1 mmol) in 10 mL THF. The reaction mixture was heated to 60 °C and kept for 24 h while being stirred. The excess of **Btl** and THF was removed by rotary evaporation, followed by vacuum drying at 70 °C for 12 h. Bis-(vinylogous urethane)-terminated **PEG** (**aPEG-Btl**) was obtained as a brownish solid (1.8 g; yield 80%).

$^{1}H$ NMR (400 MHz, DMSO-$d_6$) δ 8.48 (d, $J$ = 6.4 Hz, 1H), 4.36 (s, 1H), 4.06 – 3.99 (m, 2H), 3.51 (s, 112H), 3.27 – 3.16 (m, 2H), 1.90 (s, 3H), 1.54 – 1.43 (m, 2H), 1.38 – 1.29 (m, 2H), 0.92 – 0.88 (m, 3H).

$^{13}C$ NMR (101 MHz, DMSO-$d_6$) δ 169.71, 162.93, 81.25, 70.68, 69.33, 61.51, 42.35, 32.42, 19.87, 19.39, 14.07.

**Kinetic measurements of the hydrolysis of aPEG-Btl by in situ $^{1}$H NMR spectroscopy**

The hydrolysis of **aPEG-Btl** at varying temperatures was investigated according to a reported protocol (Y. Ma, et al. *Angew. Chem. Int. Ed.* 2023, 62, e202306188). 20 mg of **aPEG-Btl** was added to 600 μL of $D_2O$ solution in an NMR tube. The mixture was heated to different temperatures (25 °C, 45 °C, 65 °C) and the $^{1}H$ NMR spectra were measured at different time intervals (Figure S5).

The fraction of the produced **Btl** was calculated by integration of the $^{1}H$ NMR signals (Figures S5b–d), according to the equation:

Fraction **Btl** = [**Btl**]/([**aPEG-Btl**] + [**Btl**]) = Integral of proton 1'/(Integral of proton 1 + Integral of proton 1').

The extent of conversion at specific temperatures is plotted in Figure S5(e).

The rate constants ($k$) for the hydrolysis of **aPEG-Btl** with $D_2O$ were obtained by monitoring the fraction of **aPEG-Btl** in the reaction mixtures (Figures S5b–d) under pseudo-first-order conditions (large excess of $D_2O$) (Supplementary Equation 1). The activation energies $E_a$ for the small molecule reactions were determined by plotting ln($k$) vs $T^{-1}$ (Supplementary Equation 2)

Supplementary Equations (1):

ln(fraction **aPEG-Btl**) = −$k$(t), the fitting curves are shown in Figure S6(a).

Supplementary Equation (2):

$\ln(k) = \ln(A) - E_a/RT$, the fitting curve is shown in Figure S6(b).

where $T$ is the temperature expressed in Kelvin, and $R = 8.31$ J $K^{-1}$ $mol^{-1}$.

The influence of water content on the hydrolysis of **aPEG-Btl** was investigated according to the following procedures. 20 mg of **aPEG-Btl** was added to different volumes of $D_2O$ (400 μL, 500 μL, 600 μL, 800 μL) in an NMR tube. The mixture was kept at 25 °C and the $^1H$ NMR spectra were measured at different time intervals (Figure S7).

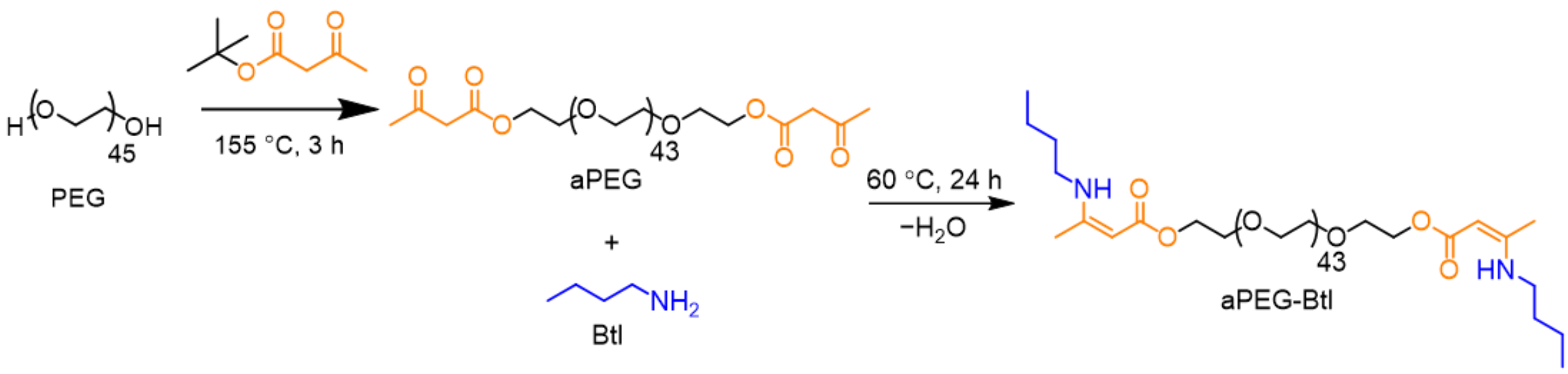


Figure S1. Synthesis of **aPEG-Btl**.

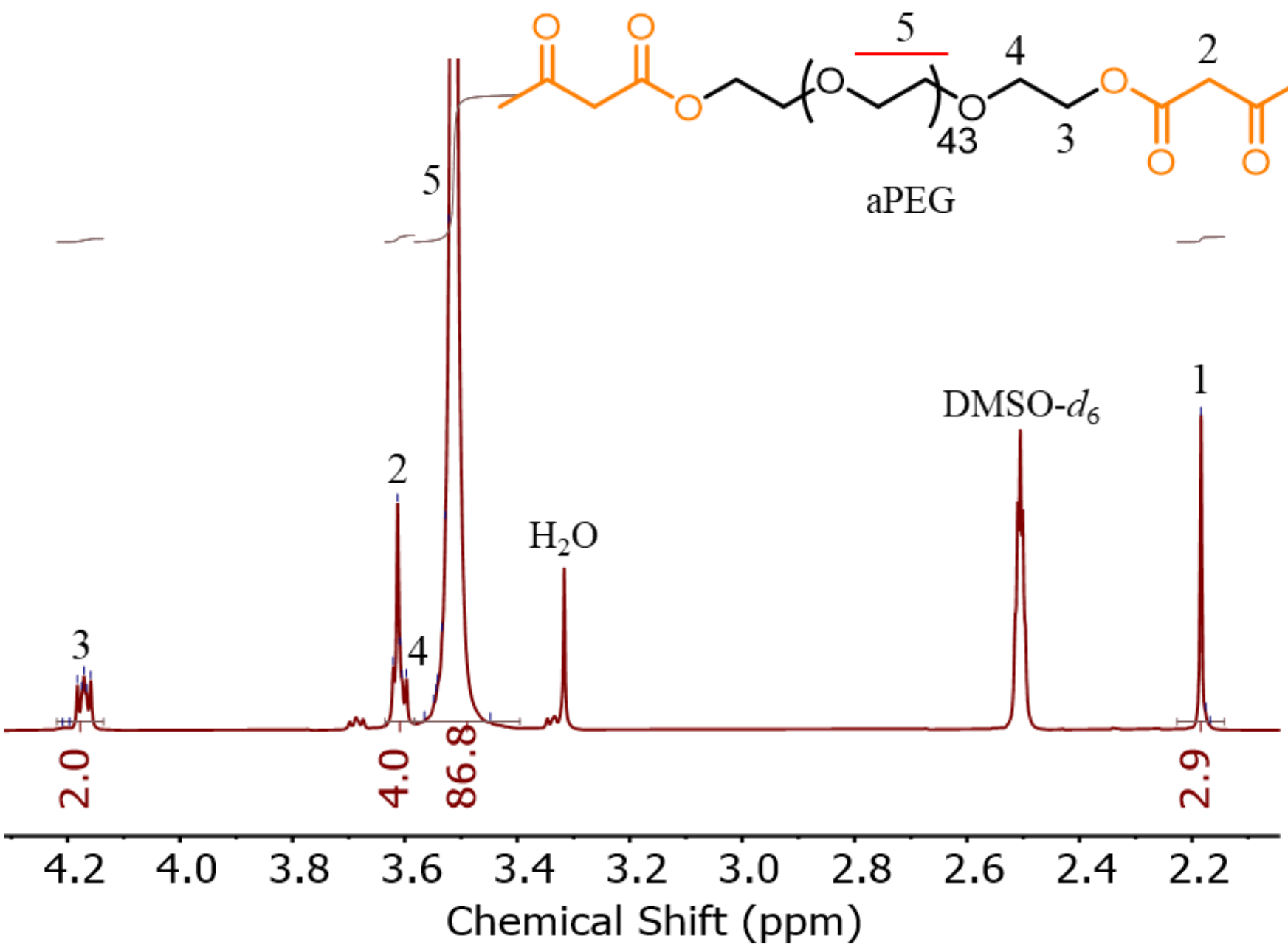


Figure S2. $^{1}H$ NMR spectrum (DMSO-$d_6$, 400 MHz) of **aPEG**.

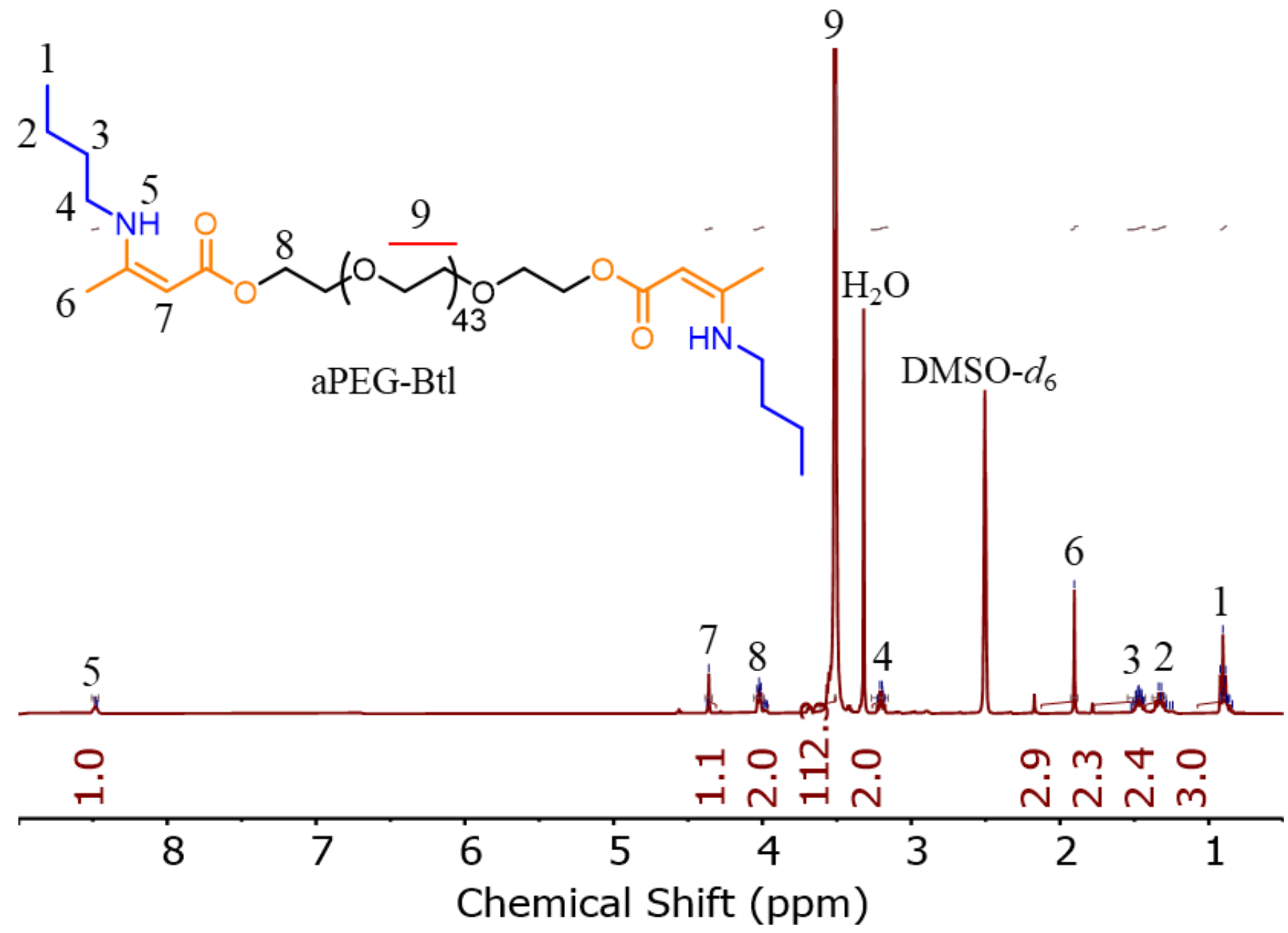

Figure S3. $^{1}H$ NMR spectrum (DMSO-$d_6$, 400 MHz) of **aPEG-Btl**.

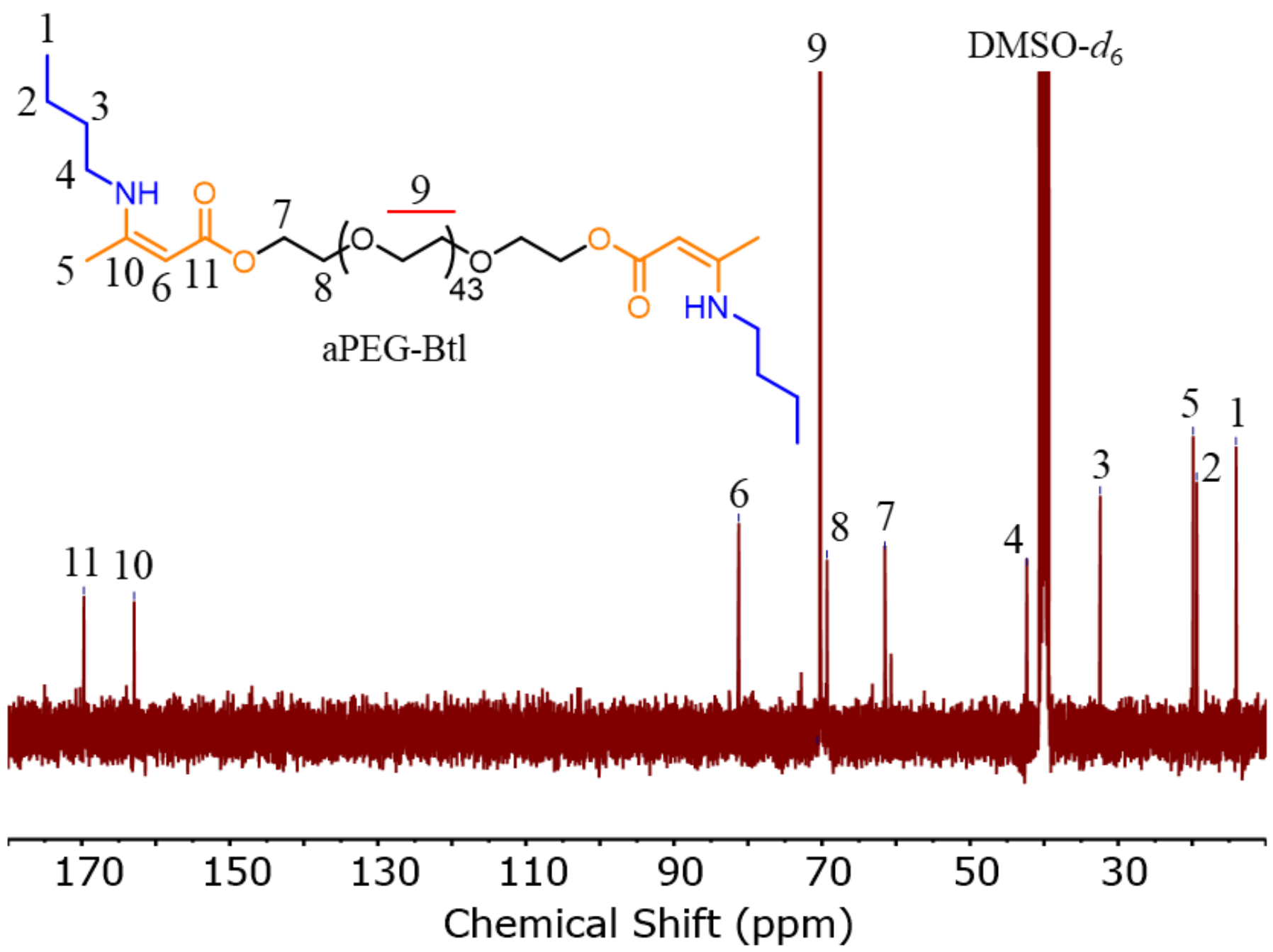

Figure S4. $^{13}C$ NMR spectrum (DMSO-$d_6$, 101 MHz) of **aPEG-Btl**.

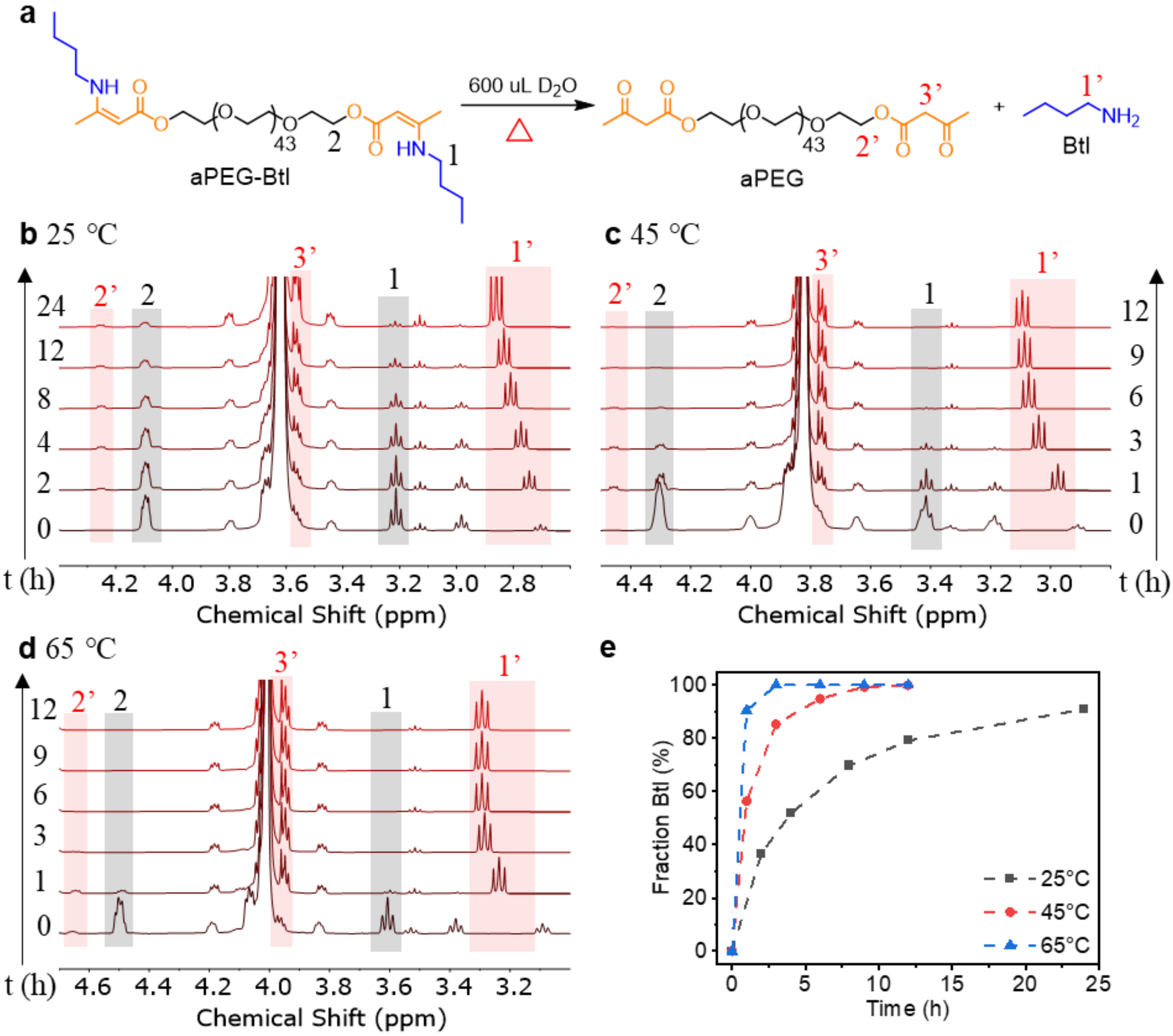


Figure S5. (a) Scheme showing the hydrolysis of **aPEG-Btl** into **aPEG** and **Btl**, with the assignment of the key protons for the integration of the $^1$H NMR signals. Evolution of the $^1$H NMR signals ($D_2O$, 400 MHz) of the reaction mixtures containing **aPEG-Btl** (20 mg) and $D_2O$ (600 µL). The samples were collected at different reaction times (as indicated), and the reactions were carried out at (b) 25 °C, (c) 45 °C, and (d) 65 °C. (e) Extent of conversion of **aPEG-Btl** into **aPEG** and **Btl** measured as a function of time at different temperatures with 600 µL of $D_2O$ present.

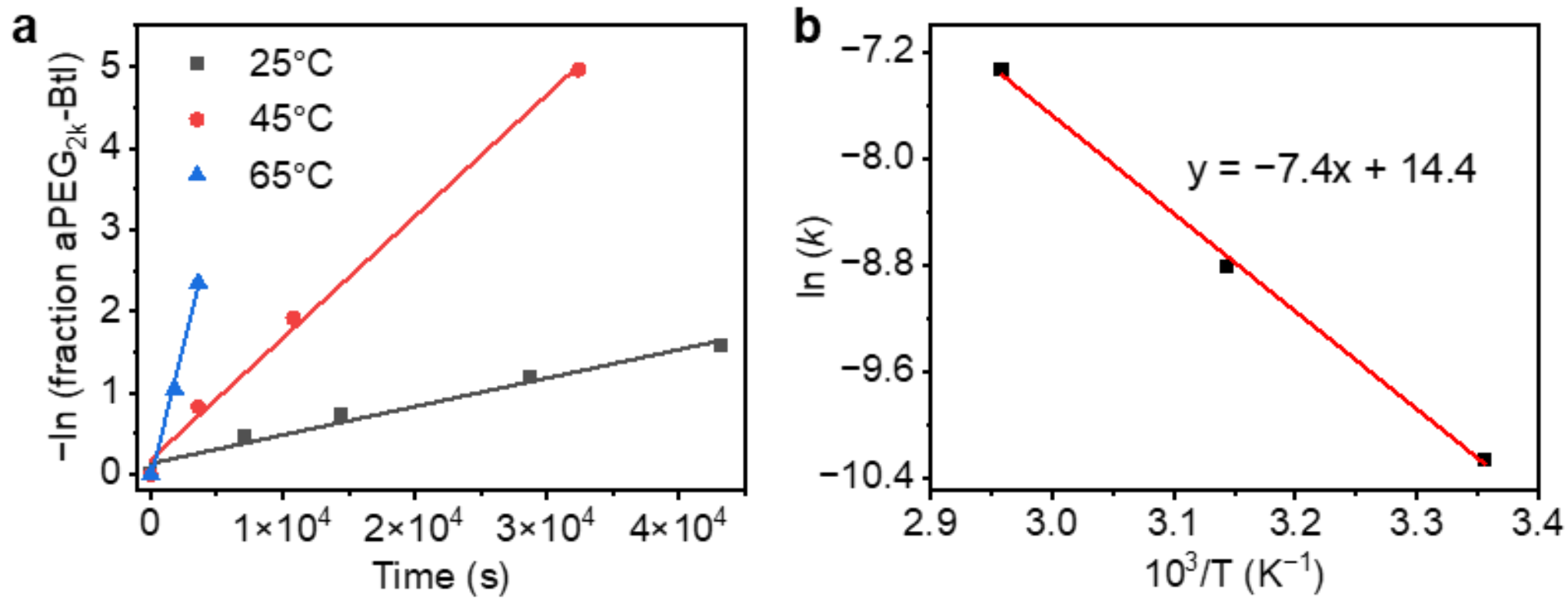


Figure S6. (a) Fitting curves and (b) Arrhenius plot for the hydrolysis between **aPEG-Btl** (20 mg) and an excess of $D_2O$ (600 μL) at different temperatures.

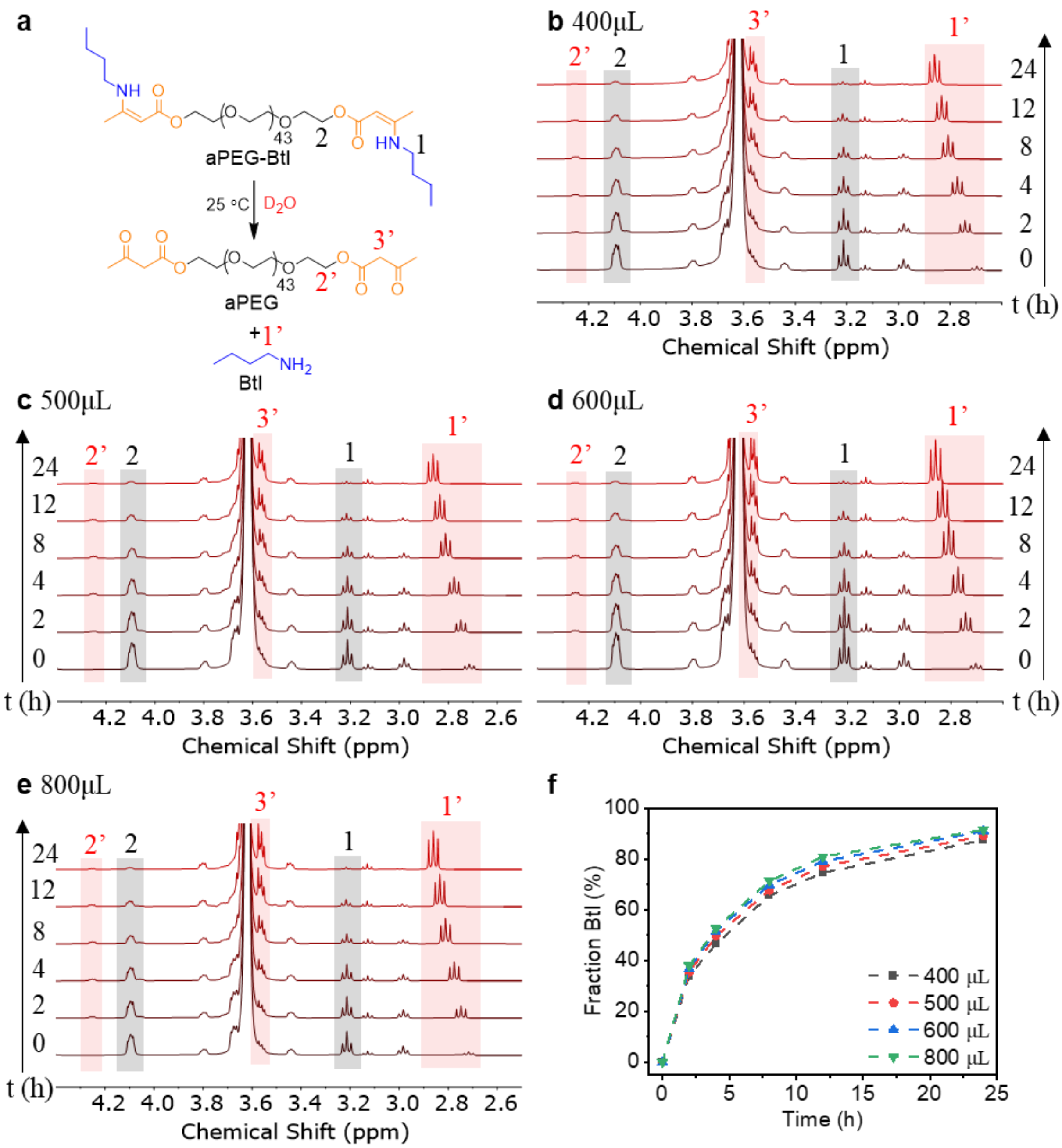


Figure S7. (a) Scheme showing the hydrolysis of **aPEG-Btl** into **aPEG** and **Btl**, with the assignment of the key protons for the integration of the $^{1}H$ NMR signals. Evolution of the $^{1}H$ NMR signals ($D_2O$, 400 MHz) of the reaction mixtures containing **aPEG-Btl** (20 mg) and different volumes of $D_2O$ (400, 500, 600, 800 μL) at 25 °C. The samples were collected at different reaction times (as indicated), and the reactions were carried out in (b) 400 μL, (c) 500 μL, (d) 600 μL, and (e) 800 μL of $D_2O$. (f) Extent of conversion of **aPEG-Btl** into **aPEG** and **Btl** measured as a function of time at 25 °C with different volumes of $D_2O$ present.

**Synthesis and hydrolysis of bis(vinylogous urethane)-terminated polypropylene glycol (PPG)**

**Synthesis of PPG bisacetoacetate (aPPG)**. As shown in Figure S8, commercial **PPG** (20 g, 10 mmol) with an $M_n$ of 2000 g mol$^{-1}$ was weighed and added into a 100 mL single-necked flask, together with a stirring bar. Then, 10 equiv. of **tBA** was added in one portion with a pipette. The flask was equipped with a condenser and a Dean–Stark apparatus to remove the *tert*-butanol generated during the reaction. The resulting mixture was heated at 155 °C for 3 h. The unreated **tBA** was removed by vacuum distillation at 100 °C for 12 h to afford **aPPG** (20.4 g; yield 93%).

$^1$H NMR (400 MHz, $CDCl_3$) δ 5.02 (dt, *J* = 11.0, 5.0 Hz, 2H), 3.46 (dtq, *J* = 20.5, 10.4, 5.3 Hz, 69H), 3.35 (dp, *J* = 13.6, 5.0 Hz, 40H), 2.20 (s, 6H), 1.18 (dd, *J* = 6.5, 2.4 Hz, 6H), 1.07 (dd, *J* = 6.1, 3.3 Hz, 101H).

**Synthesis of bis(vinylogous urethane)-terminated PPG**. As shown in Figure S8, in a 25 mL two-necked flask equipped with a reflux condenser and a magnetic stir bar, **Btl** (0.73 g, 10 mmol) was combined with **aPPG** (2.2 g, 1 mmol) in 10 mL THF. The reaction mixture was heated to 60 °C and kept for 24 h while being stirred. The excess of **Btl** and THF was removed by rotary evaporation, followed by vacuum drying at 70 °C for 12 h. Bis-(vinylogous urethane)-terminated **PPG** (**aPPG-Btl**) was obtained as a yellowish liquid (2.1 g; yield 90%).

$^1$H NMR (400 MHz, $CDCl_3$) δ 8.54 (s, 2H), 5.04 – 4.92 (m, 2H), 4.43 (s, 2H), 3.64 – 3.32 (m, 108H), 3.19 (q, *J* = 6.8 Hz, 4H), 1.90 (s, 6H), 1.57 – 1.52 (m, 4H), 1.42 – 1.37 (m, 4H), 1.22 (d, *J* = 6.4 Hz, 6H), 1.14 (dd, *J* = 5.9, 3.3 Hz, 101H), 0.93 (t, *J* = 7.3 Hz, 6H).

$^{13}$C NMR (101 MHz, $CDCl_3$) δ 170.05, 161.92, 82.10, 75.53, 74.00, 72.93, 67.17, 42.74, 32.50, 20.02, 19.39, 17.48, 13.78.

**Kinetic measurements of the hydrolysis of PPG-Btl by in situ $^1$H NMR spectroscopy**

**aPPG-Btl** features high hydrophobicity and can not dissolve in water. A two-phase solution system was then adopted following a reported protocol (Y. Ma, et al., *Macromolecules* 2025, 58, 1923–1934). The mixture solution comprised 4 mL 1M HCl or $H_2O$ as the upper phase and 4 mL of $CDCl_3$ in the bottom phase (Figure S12b). Specifically, 200 mg of **aPTHF$_{2k}$-Btl** was first dissolved in 4 mL of $CDCl_3$ in a 20 mL vial. 4 mL 1M HCl or $H_2O$ was then added and suspended above the $CDCl_3$ solution. The resulting mixture was kept at 25 °C while being stirred at 400 rpm. At different reaction times, 0.5 mL aliquots of the $CDCl_3$ solution phase were taken out with a syringe and transferred to an NMR tube for measurements.

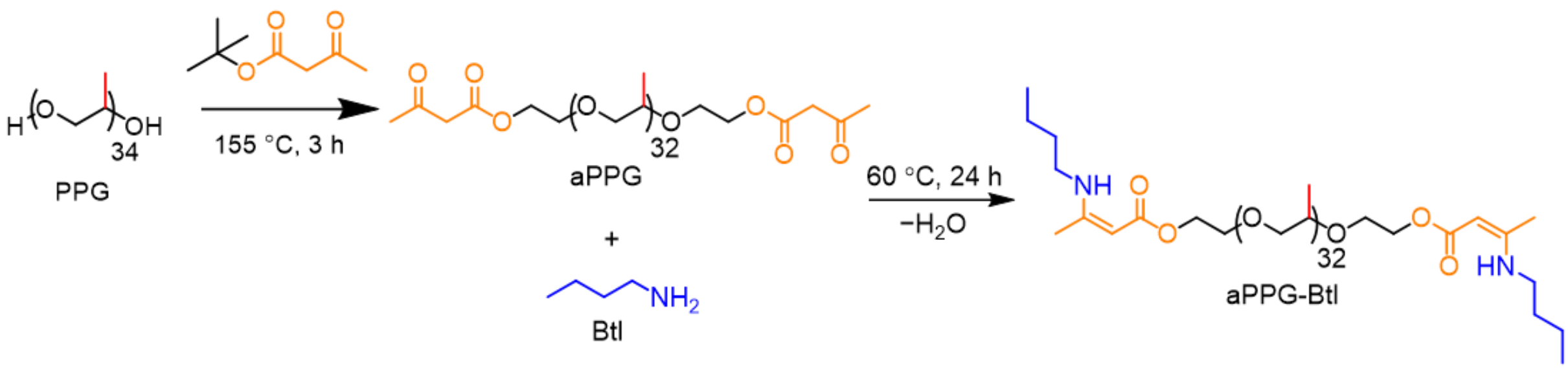


Figure S8. Synthesis of **aPPG-Btl**.

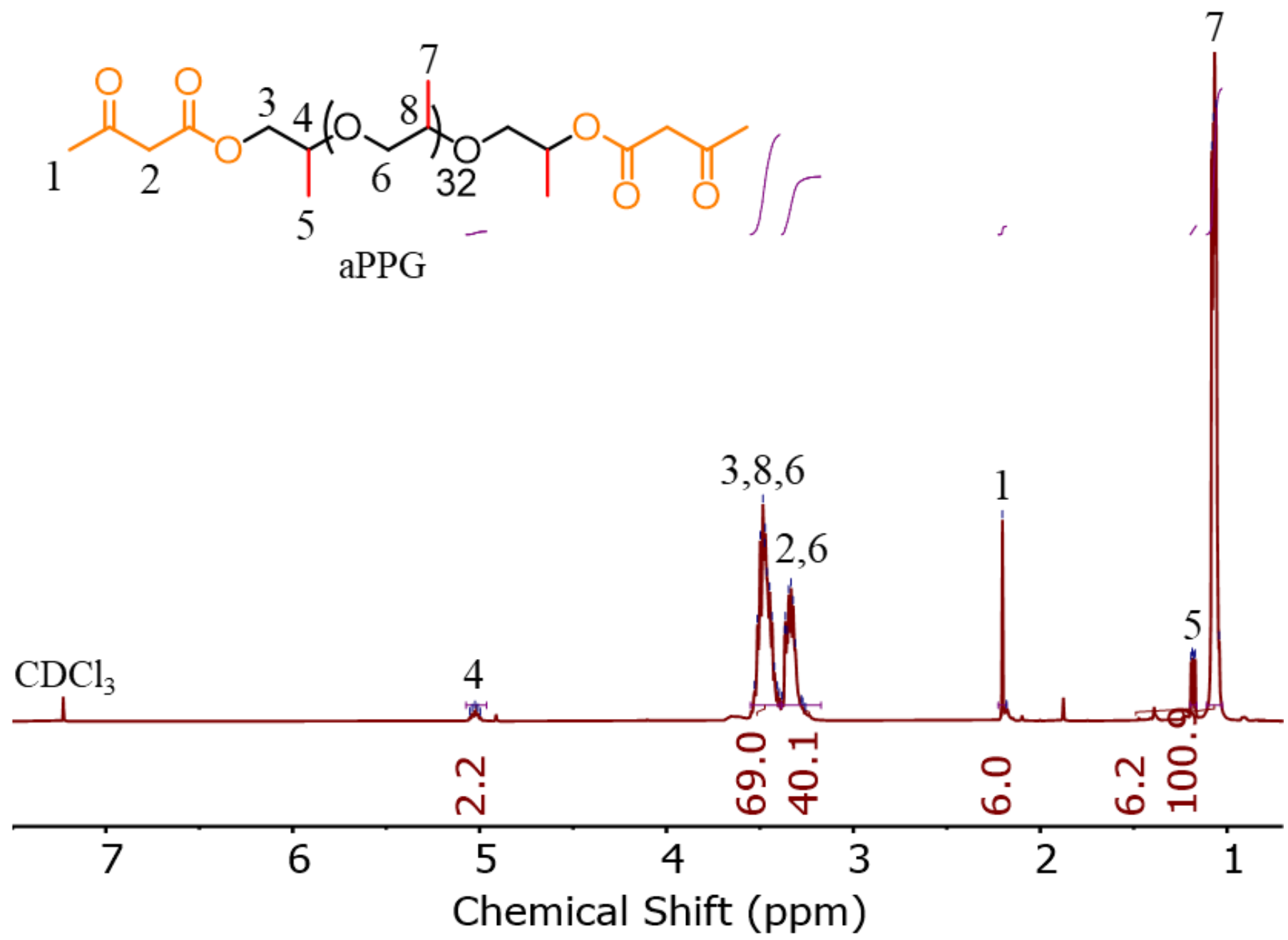


Figure S9. $^{1}$H NMR spectrum ($CDCl_3$, 400 MHz) of **aPPG**.

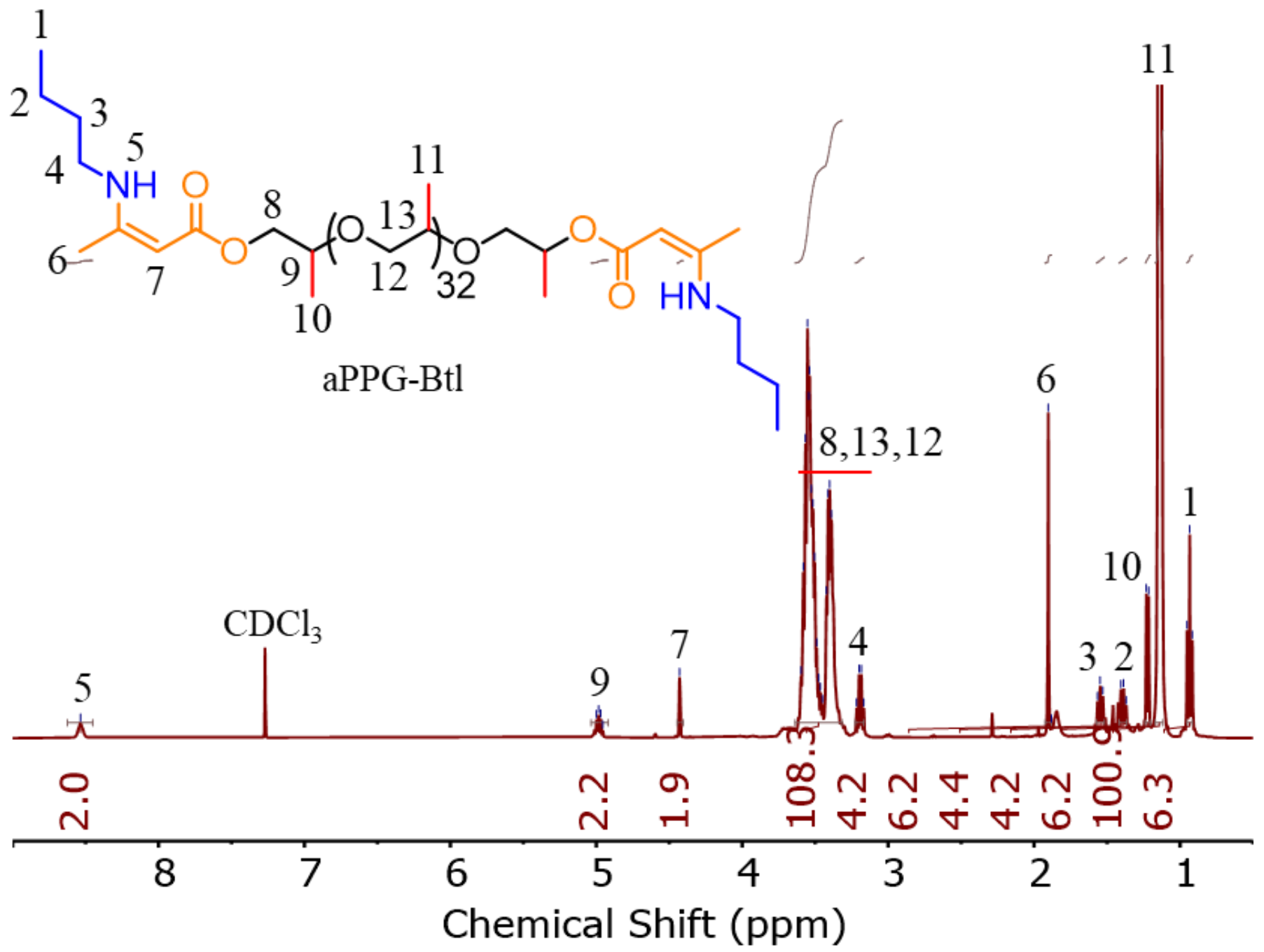


Figure S10. $^{1}$H NMR spectrum ($CDCl_3$, 400 MHz) of **aPPG-Btl**.

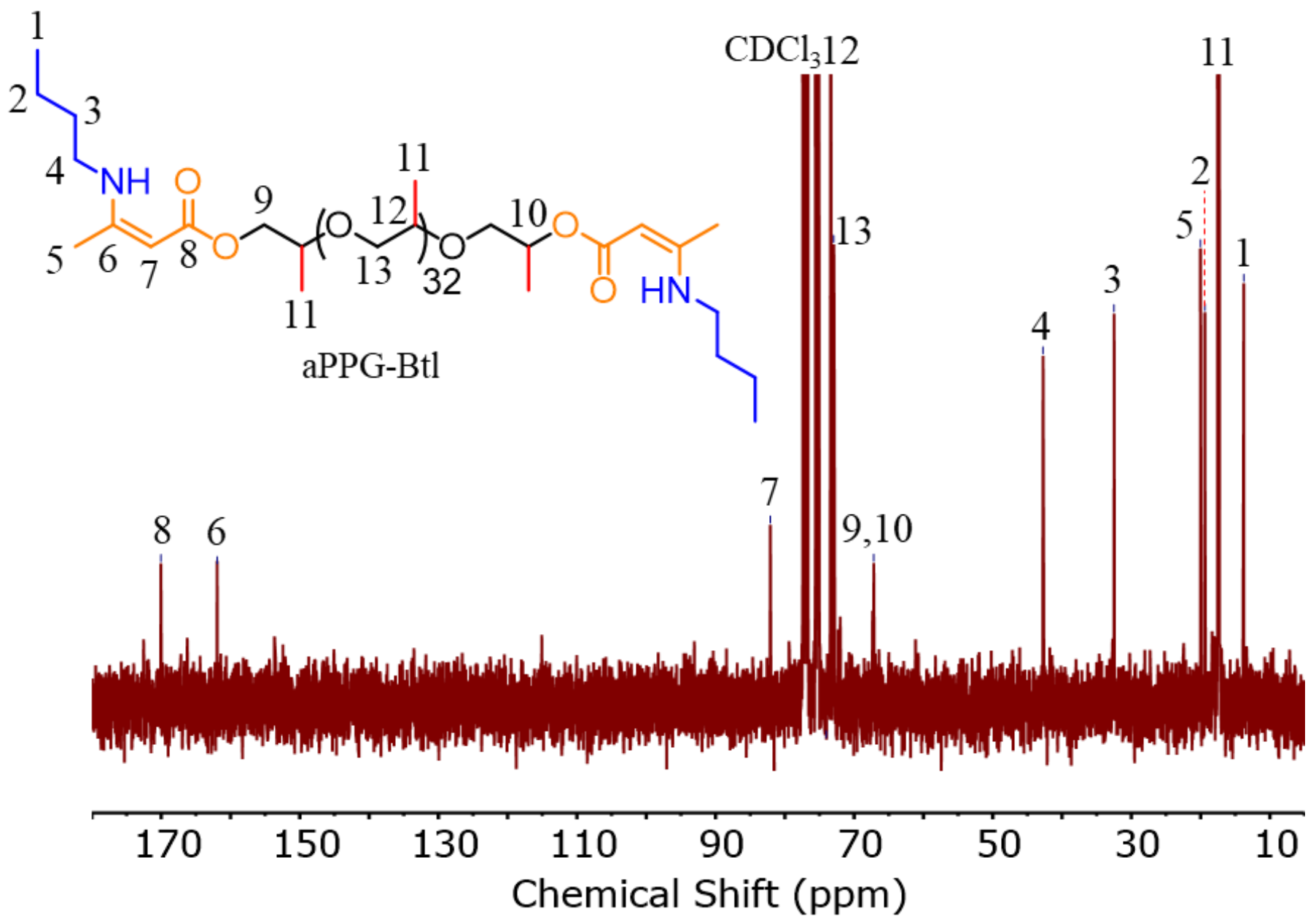


Figure S11. $^{13}$C NMR spectrum ($CDCl_3$, 101 MHz) of **aPPG-Btl**.

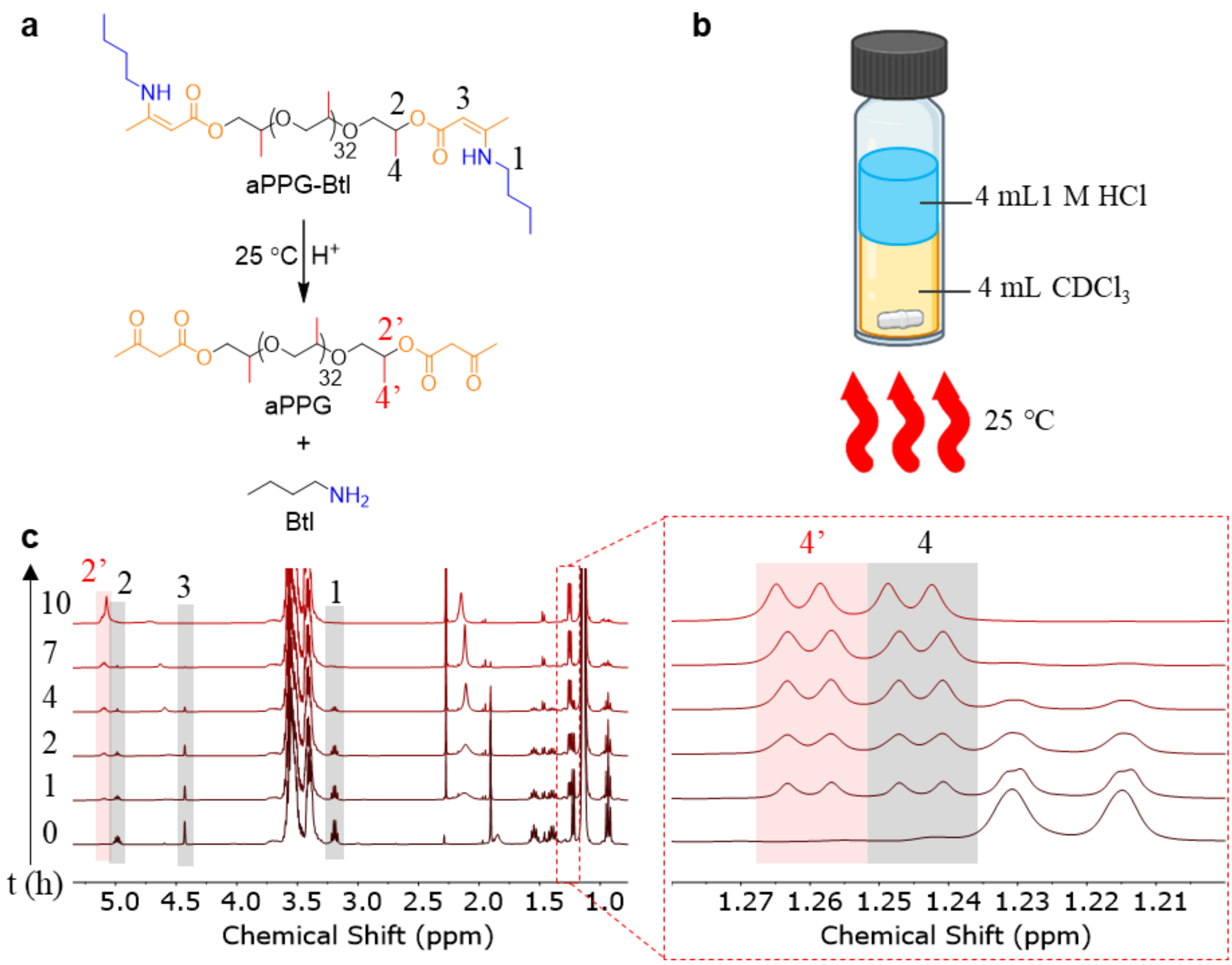


Figure S12. (a) Scheme showing the dissociation of **aPPG-Btl** into **aPPG** and **Btl**, with the assignment of the key protons shown in the $^1$H NMR spectra. (b) Schematic illustration showing a two-phase mixture solution comprising 4 mL of 1M HCl (upper phase) and 4 mL of $CDCl_3$ (bottom phase) at 25 °C. (c) Evolution of the $^1$H NMR signals ($CDCl_3$, 400 MHz) of the hydrolysis reaction of **aPPG-Btl** (200 mg) in the 1M HCl–$CDCl_3$ mixture solution with the composition illustrated in (b).

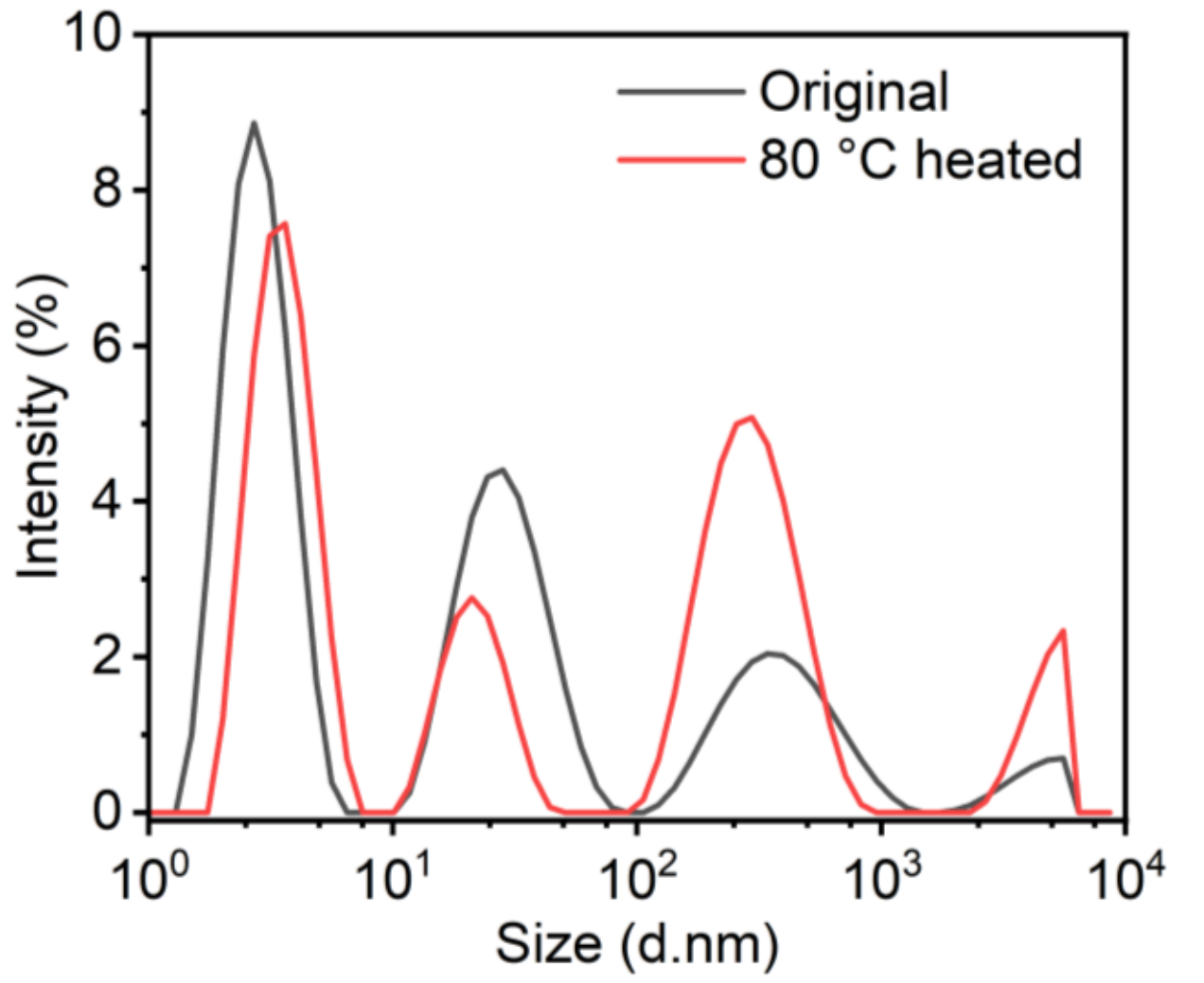


Figure S13. Size distribution of the original **BSA** and the **BSA** after being heated at 80 ℃ for 2 h, measured in water at a concentration of 2 mg mL$^{-1}$ by DLS. The average hydrodynamic diameter (*D*) of the original **BSA** is 10.4 ± 2.7 nm, with a PDI of 0.44 ± 0.04; The average *D* of 80 ℃-treated **BSA** is 25.3 ± 4.9 nm, with a PDI of 0.5 ± 0.02.

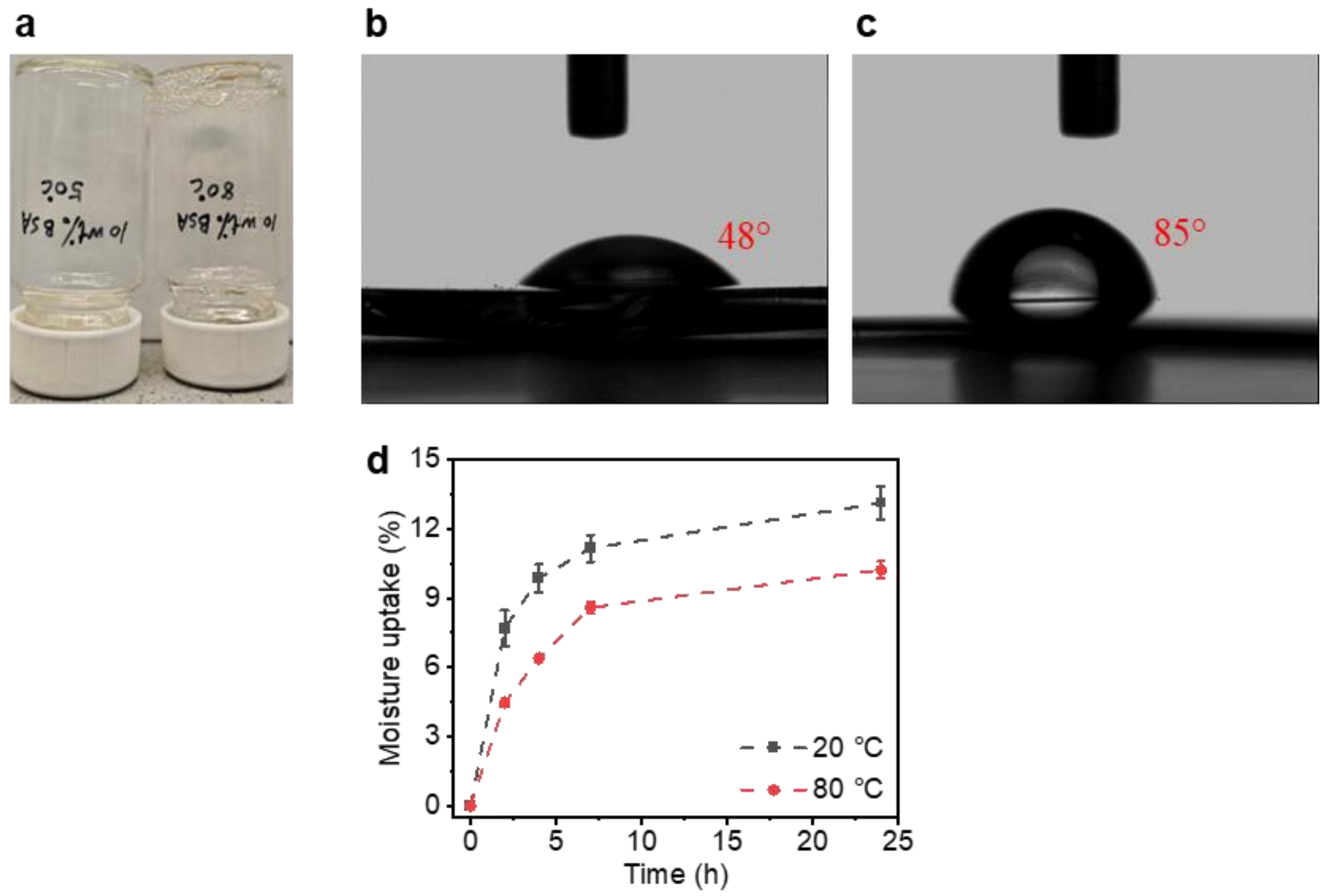


Figure S14. (a) Photograph showing an inverted small vial containing 10 wt% **BSA** aqueous solution after being treated at 50 (left) or 80 °C (right) for 2 days. Water contact angle images of **BSA** films obtained through thermal treatment at (b) 50 °C or (c) 80 °C for 2 days, with the second day treated under vacuum. (d) Moisture uptake in 75 %RH as a function of time of **BSA** samples after being heated at 20 °C or 80 °C for 2 days.

**Synthesis and recycling of VU-cross-linked bovine serum albumin (BSA-aPEG)**

**Synthesis of BSA-aPEG**. In a typical procedure to synthesize **BSA-aPEG** films, 2 g **BSA** was dissolved in 10 mL $H_2O$ in a 20 mL vial. Then, a different amount of **aPEG** was added to the mixture. The resulting solution was stirred for 30 min at room temperature, and then poured into a Teflon mold that was covered with an aluminium foil. The mixture was heated at 80 °C for 24 h in a drying oven (no vacuum) before vacuum drying at 80 °C for another 24 h. An off-yellowish film was obtained, which was labelled as **BSA-xaPEG**. **x** represents the weight ratio of **aPEG** to **BSA** as 10, 20, 30 in percentage. The molecular weight of **BSA** is 66.5 kDa, comprising 59 lysine (Lys) residues and 23 arginine (Arg) residues per chain. 2 g BSA could theoretically consume 2.67 g of **aPEG** given the complete reaction of all primary amino groups (E3S Web of Conferences 2021, 309, 01086). Thus, the theoretical conversions of amino groups to **VU** bonds are 7.5 % for **BSA-10aPEG**, 15.0% for **BSA-20aPEG**, and 22.5% for **BSA-30aPEG**.

A control protein film that does not undergo significant thermal denaturation was also prepared. Instead of being treated at 80 °C, the mixture solution of **BSA** and 20 wt% of **aPEG** was poured into a Teflon mold, and heated at 50 °C for 48 h initially without vacuum treatment before vacuum drying at such a temperature for another 24 h. The obtained film was labelled **BSA-20aPEG-50 °C**.

**Recycling of BSA-20aPEG(−50 °C)**. For the recycling of **BSA-20aPEG-50 °C**, as shown in Figure S19(a), small pieces of film (0.2 g) were immersed in 5 mL deionized $H_2O$ and stirred for 24 h. It formed a clear solution. Then, the solution was poured into an excessive amount of ethanol (ca. 100 mL) and it led to precipitation. The precipitated solid was separated by vacuum filtration and washed with ethanol three times. After vacuum drying at room temperature overnight, 0.15 g of **BSA** powder was obtained in 90% yield. The liquid solution was combined and first subjected to rotary evaporation to remove most solvent before vacuum drying at 50 °C overnight to obtain 21 mg solid (yield 64%).

The recycling of **BSA-20aPEG** followed a different protocol. First, small pieces of film (0.2 g) were immersed in 5 mL 0.1 M HCl and stirred for 24 h. After that, the solution was precipitated by ethanol. The solid produced was separated by vacuum filtration and washed with $H_2O$ three times. After vacuum drying at 50 °C overnight, 148 mg of **BSA** powder was recovered in 89% yield. The liquid was combined, and first neutralized by 1 M $NaHCO_3$ before it was dried by rotary evaporation to remove $H_2O$. The resulting solid was extracted with $CDCl_3$ for three times. The solution obtained was first dried by rotary evaporation, followed by vacuum drying at 60 °C overnight to obtain 25 mg solid (yield 83%). It was identified as **PEG** by $^1H$ NMR analysis. The investigation of its formation is shown in Figure S22.

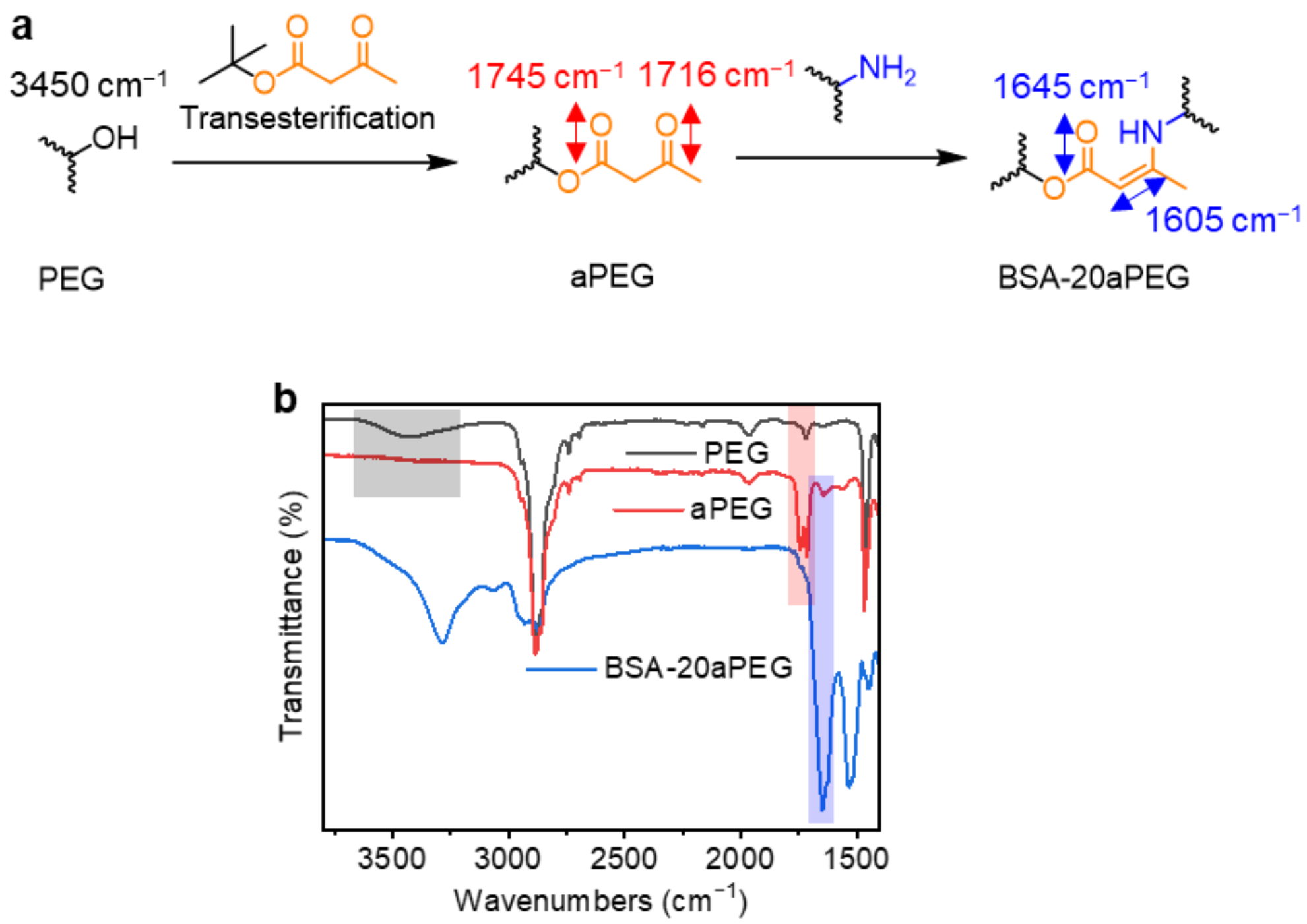


Figure S15. (a) Synthesis of **BSA-20aPEG**. (b) FTIR spectra of **PEG**, **aPEG**, and **BSA-20aPEG**.

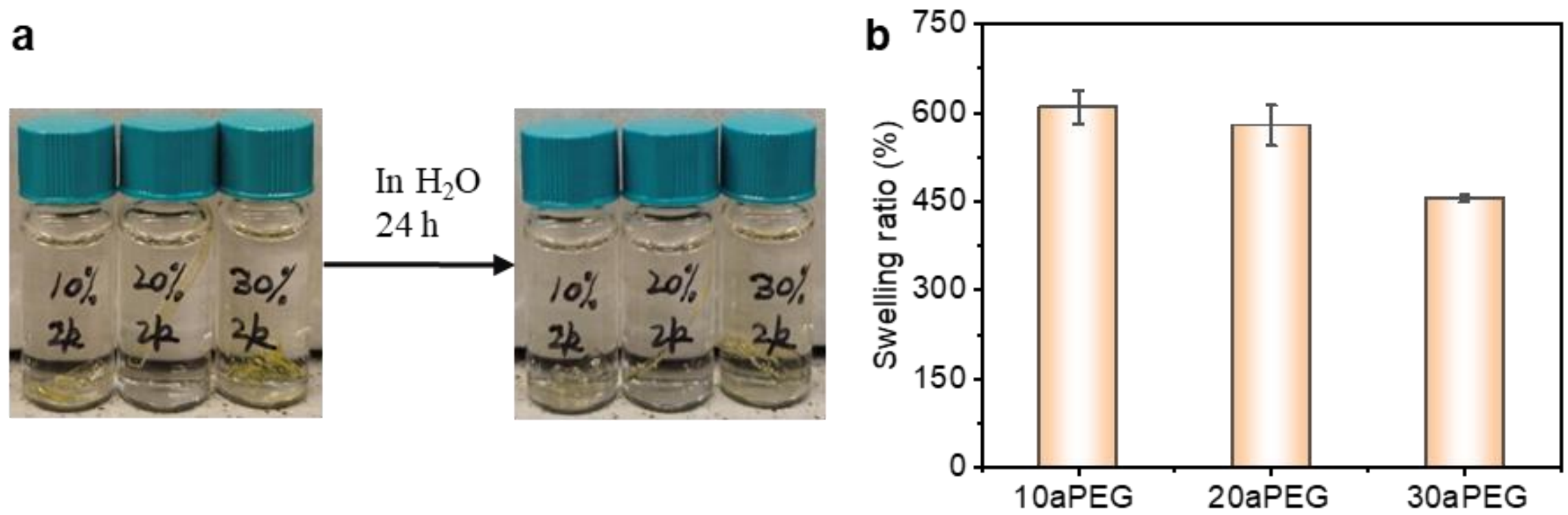


Figure S16. (a) Photographs of **BSA-xaPEG** films dispersed in $H_2O$ before (left) and after (right) 1 day incubation at room temperature. (b) Swelling ratio of **BSA-xaPEG** films.

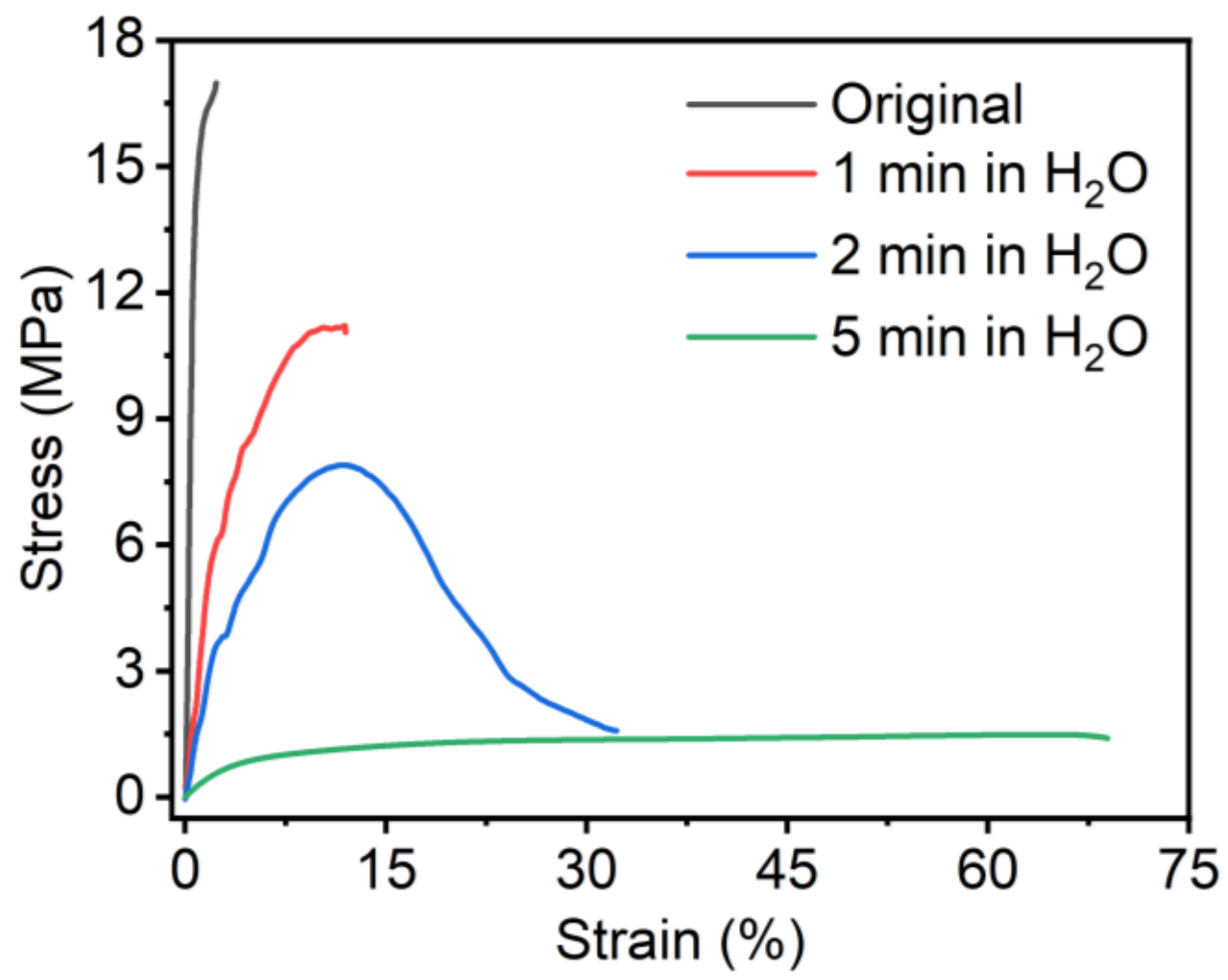


Figure S17. Stress–strain curves of the **BSA-20aPEG** films, measured after the immersion in $H_2O$ for different times (as indicated).

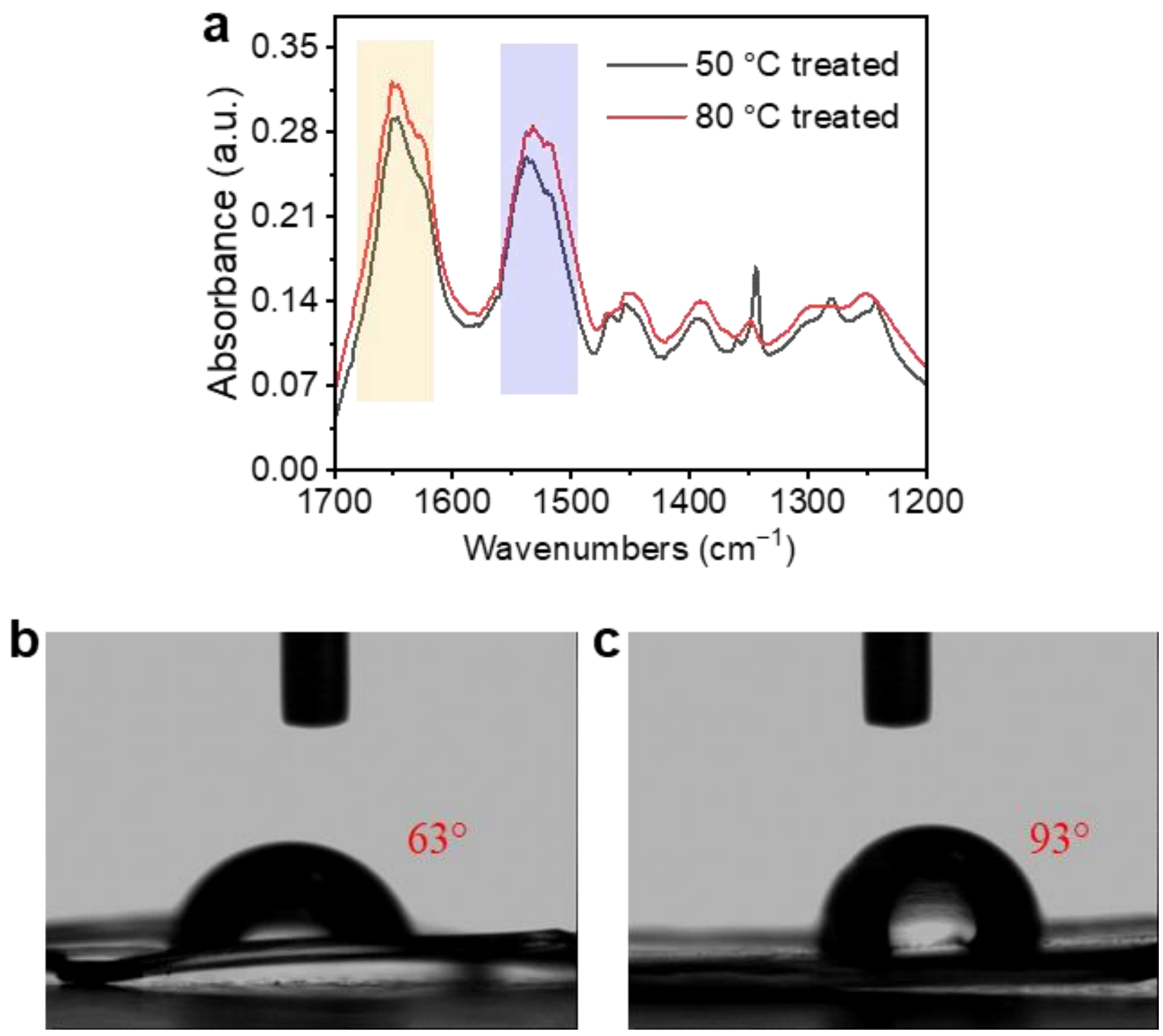


Figure S18. (a) FTIR spectra of **BSA-20aPEG** obtained through thermal treatment at 50 or 80 °C. Water contact angle images of (b) **BSA-20aPEG-50 °C** and (c) **BSA-20aPEG-80 °C**.

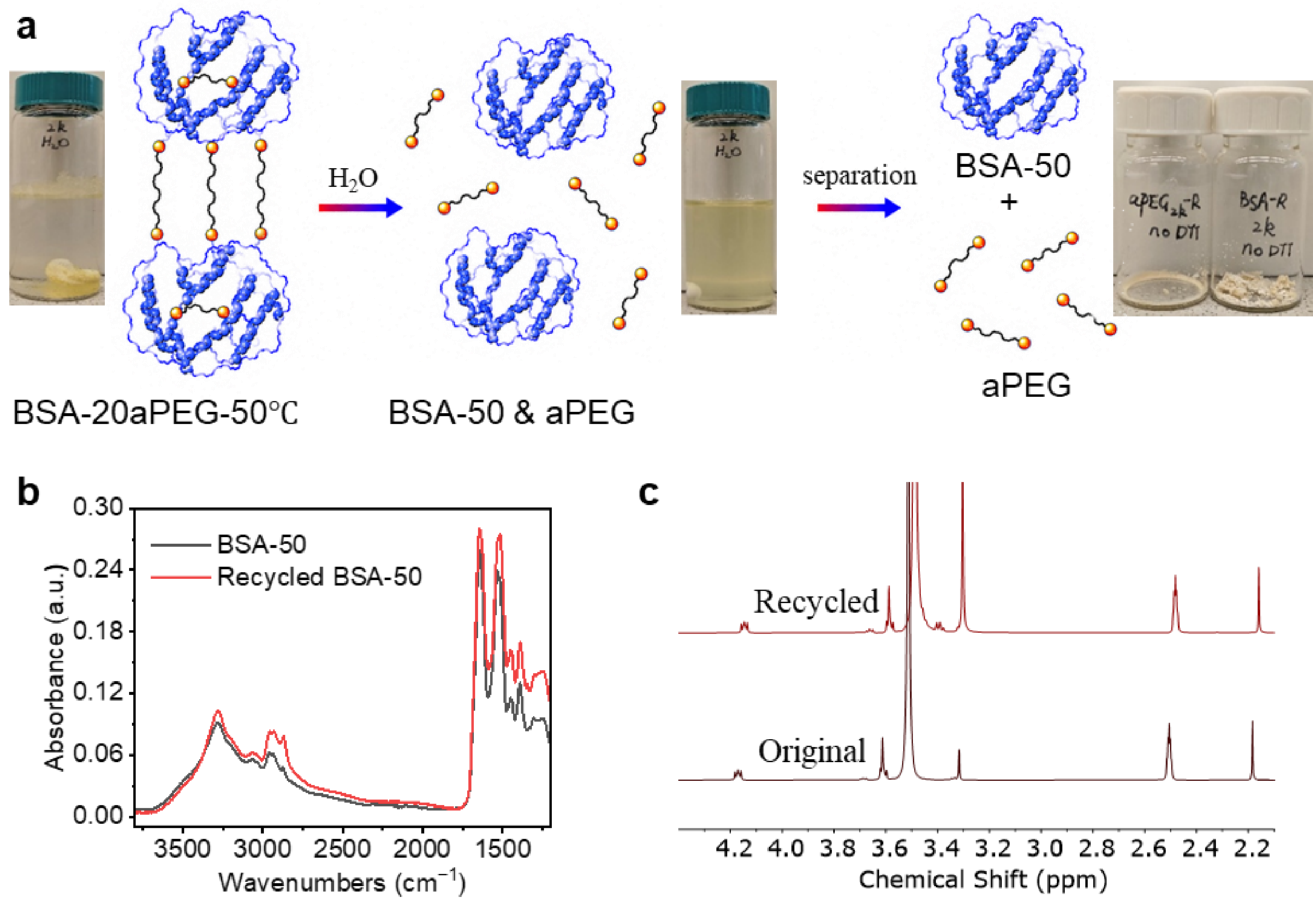


Figure S19. (a) Illustration of the hydrolysis of **BSA-20aPEG-50 °C**, followed by chemical separation to recover both **BSA-50** and **aPEG**. (b) FTIR spectra of the **BSA** after being treated at **50 °C** for 2 days and the recovered **BSA-50**. (c) $^1$H NMR spectrum (DMSO-$d_6$, 400 MHz) of the original and recycled **aPEG**.

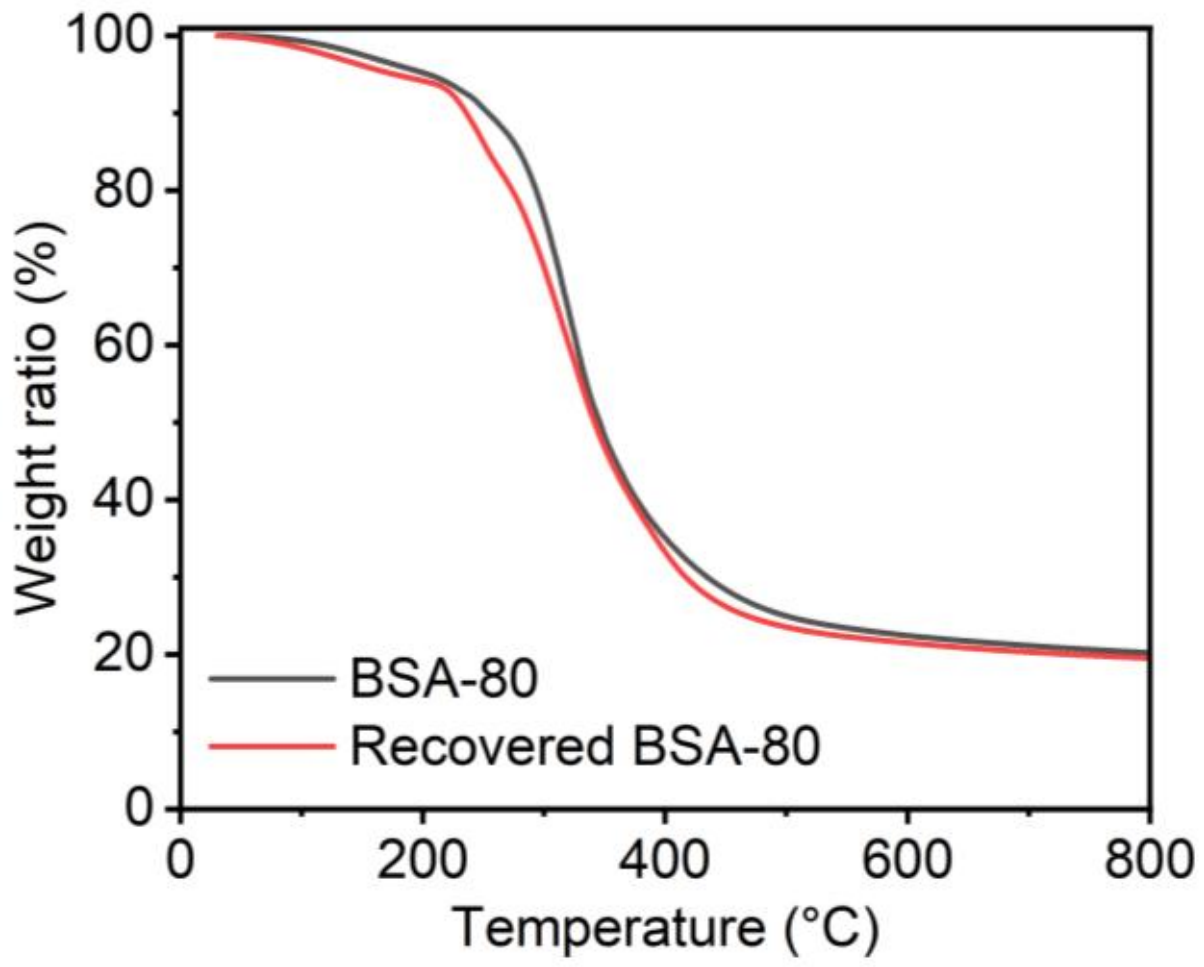


Figure S20. Thermogravimetric curves of the original BSA-80 and the chemically recycled BSA-80 by acid.

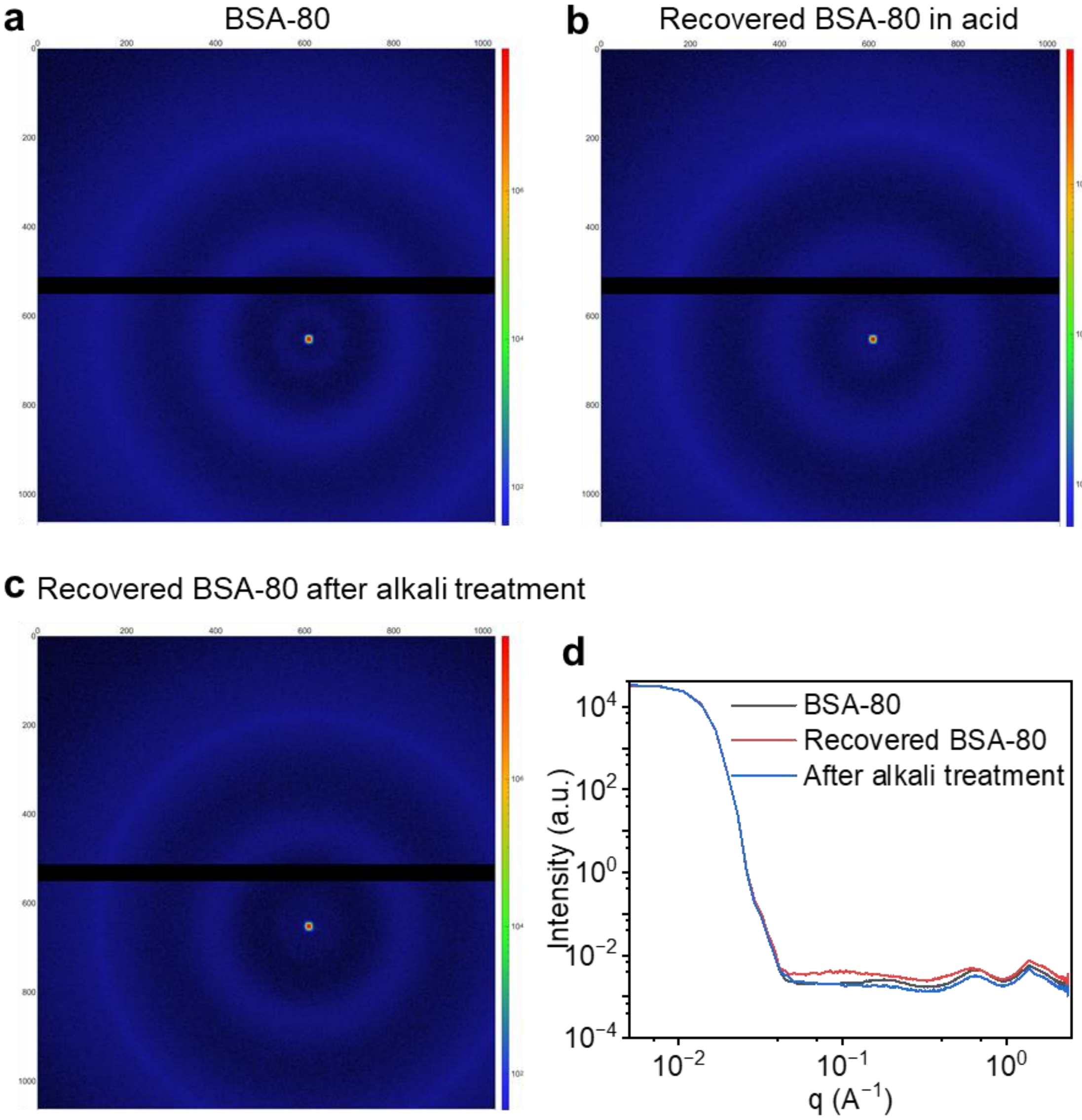


Figure S21. 2D-SAXS/WAXS patterns of (a) the original BSA-80, (b) the chemically recycled BSA-80 by acid, and (c) the neutralized recovered BSA-80 by 0.1 M $NaHCO_3$. (d) 1D-SAXS/WAXS profiles of the original BSA-80, the chemically recycled BSA-80 by acid, and the neutralized recovered BSA-80 by 0.1 M $NaHCO_3$ aqueous solution.

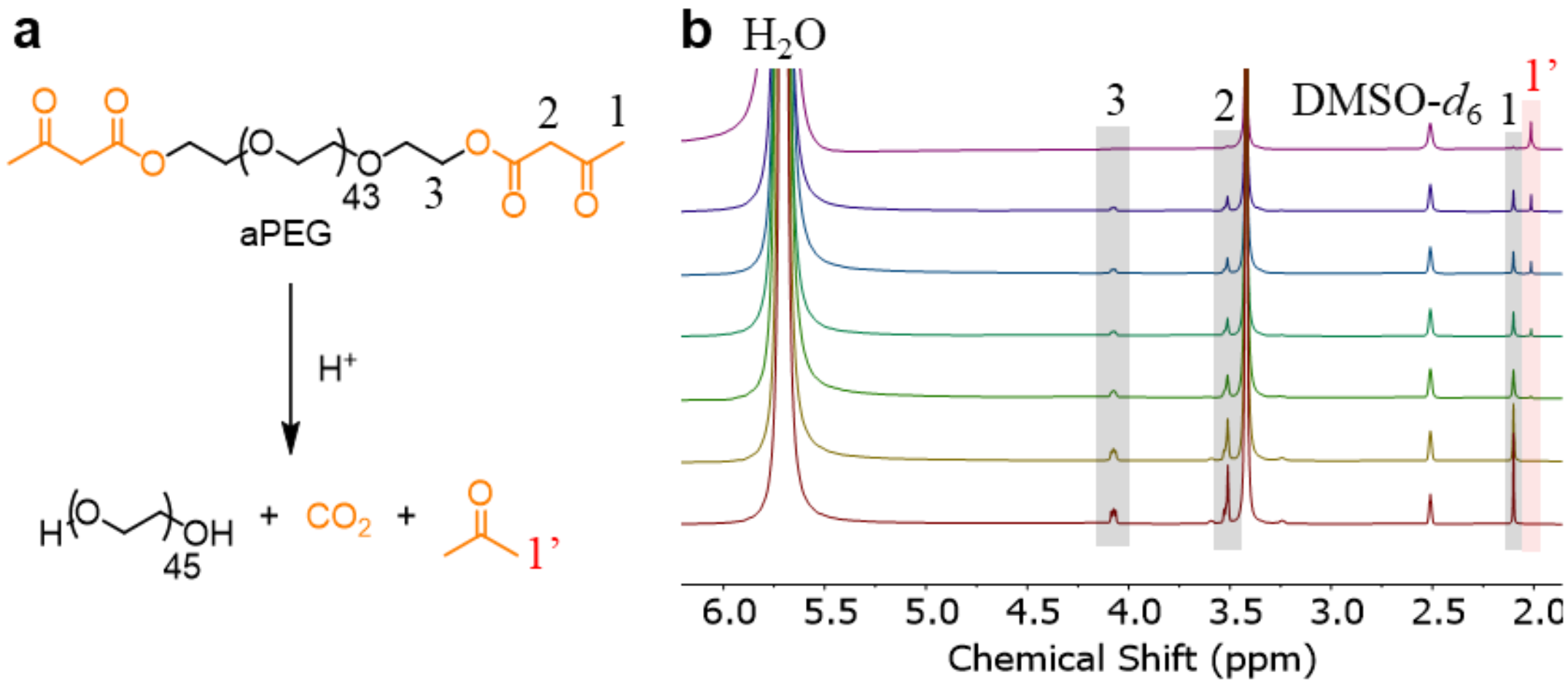


Figure S22. Scheme showing the dissociation of **aPEG** into **PEG**, acetone, and $CO_2$, with the assignment of the key protons shown in the $^1$H NMR spectra. Evolution of the $^1$H NMR signals (DMSO-$d_6$, 400 MHz) of the dissociation reaction of **aPEG** (20 mg) in the mixture solution (500 uL) of DMSO-$d_6$/1M HCl at a volume ratio of 9/1.
